# Engineered bacterial host for genetic encoding of physiologically stable protein nitration

**DOI:** 10.3389/fmolb.2022.992748

**Published:** 2022-10-24

**Authors:** Nikolaj G. Koch, Tobias Baumann, Jessica H. Nickling, Anna Dziegielewski, Nediljko Budisa

**Affiliations:** ^1^ Bioanalytics Group, Institute of Biotechnology, Technische Universität Berlin, Berlin, Germany; ^2^ Biocatalysis Group, Institute of Chemistry, Technische Universität Berlin, Berlin, Germany; ^3^ Chemical Synthetic Biology Group, Department of Chemistry, University of Manitoba, Winnipeg, MB, Canada

**Keywords:** elastin-like polypeptides, MjTyrRS, ONBY, lambda red recombineering, Keio collection, nitroreduction, mussel-inspired adhesives, genetic code expansion

## Abstract

Across scales, many biological phenomena, such as protein folding or bioadhesion and cohesion, rely on synergistic effects of different amino acid side chains at multiple positions in the protein sequence. These are often fine-tuned by post-translational modifications that introduce additional chemical properties. Several PTMs can now be genetically encoded and precisely installed at single and multiple sites by genetic code expansion. Protein nitration is a PTM of particular interest because it has been associated with several diseases. However, even when these nitro groups are directly incorporated into proteins, they are often physiologically reduced during or shortly after protein production. We have solved this problem by using an engineered *Escherichia coli* host strain. Six genes that are associated with nitroreductase activity were removed from the genome in a simple and robust manner. The result is a bacterial expression host that can stably produce proteins and peptides containing nitro groups, especially when these are amenable to modification. To demonstrate the applicability of this strain, we used this host for several applications. One of these was the multisite incorporation of a photocaged 3,4-dihydroxyphenylalanine derivative into Elastin-Like Polypeptides. For this non-canonical amino acid and several other photocaged ncAAs, the nitro group is critical for photocleavability. Accordingly, our approach also enhances the production of biomolecules containing photocaged tyrosine in the form of ortho-nitrobenzyl-tyrosine. We envision our engineered host as an efficient tool for the production of custom designed proteins, peptides or biomaterials for various applications ranging from research in cell biology to large-scale production in biotechnology.

## 1 Introduction

Over the past two decades, genetic code expansion has evolved into a powerful technique to genetically introduce non-canonical amino acids (ncAAs) into proteins and peptides (Liu and Schultz, 2010; Mukai et al., 2017; de la Torre and Chin, 2021). More than 200 ncAAs with a broad repertoire of chemistries and new-to-nature functionalities have been incorporated by various routes (Wan et al., 2014; Dumas et al., 2015; Koch et al., 2021; Pagar et al., 2021). The most commonly used method is site-specific stop codon suppression (SCS) *in vivo*, using bacterial hosts. A key application is to genetically encode post-translational modifications (PTMs) to elucidate their effects on protein activity (Hoppmann et al., 2017; Luo et al., 2017; Young and Schultz, 2018; Zhu et al., 2019; Italia et al., 2020). In particular, mimicking protein nitration is of great interest since this PTM has been linked to several diseases (Neumann et al., 2008; Porter et al., 2019; Jang et al., 2020). However, a major challenge in obtaining these recombinant proteins remains: Especially in bacterial hosts, molecules containing nitro groups are often enzymatically reduced to hydroxylamino and/or amino derivatives (Copp et al., 2017). This side chain modification also occurs in a variety of ncAAs and was also observed in our previous study in which a photo-caged derivative of 3,4-dihydroxyphenylalanine (Dopa) was genetically encoded (Nguyen et al., 2014; Ren et al., 2015; Hauf et al., 2017; Baumann et al., 2019; Böcker et al., 2019). Dopa moieties play a crucial role in wet surface adhesion of marine mussels mediated by foot proteins (Ahn et al., 2015; Maier et al., 2015; Seo et al., 2015; Waite, 2017; Budisa and Schneider, 2019).

**FIGURE 1:**
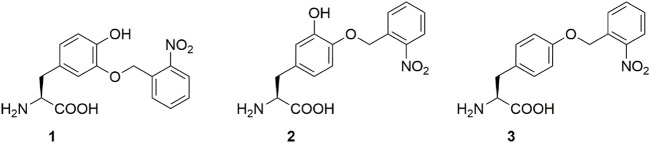
Photocaged non-canonical amino acids used in this study. *meta*-(*ortho*-(2-nitrobenzyl))-3,4-dihydroxyphenylalanine (*m*-oNB-Dopa, **1**), *para*-(*ortho*-(2-nitrobenzyl))-3,4-dihydroxyphenylalanine (*p*-oNB-Dopa, **2**) and *ortho*-nitro-benzyl-tyrosine (ONBY, **3**).

A long-standing goal of our laboratory is to harness these properties and to engineer biomaterial-based wet-adhesion agents that can be used, for example, for *in vivo* tissue or wound healing and bone regeneration (Haller et al., 2012; Kaushik et al., 2015; Kim et al., 2021). Unfortunately, the tendency of Dopa to oxidize to its quinone moiety under normal (oxidative) protein production and purification conditions makes handling Dopa containing proteins challenging. One solution was to genetically encode a Dopa analog, *meta*-(*ortho*-(2-nitrobenzyl))-3,4-dihydroxy-phenylalanine (*m*-oNB-Dopa (**1**), Figure 1) which is resistant to oxidization. Moreover, the protecting group is photocleavable, conferring spatiotemporal control over the adhesion mechanism (Hauf et al., 2017). Unfortunately, as mentioned above, the nitro group is reduced in bacterial production hosts, which drastically reduces photocleavability.

Despite the fascinating properties of mussel adhesion proteins, their recombinant production in high yields is a general obstacle. The main reasons for this are probably the low protein solubility and the low in-frame SCS efficiency during ribosomal synthesis (Hauf et al., 2017). Considering their natural purpose and function, it makes sense that mussels produce adhesion proteins that are insoluble in water-based solutions. Moreover, the incorporation efficiency of ncAAs depends on the protein scaffold and sequence context (Pott et al., 2014; Chemla et al., 2018; Schwark et al., 2018). We thus concluded that these problems could be circumvented in one step by switching the target protein scaffold from mussel proteins to elastin-like polypeptides (ELPs). ELPs are biopolymers composed of the repeating pentapeptide sequence (VPGXG)_n_ (see Supplementary Material for details). Hallmark of the ELP structure are tandem repeats with repeating proline (conferring stiffness) and glycine (conferring flexibility), while the “host” position X tolerates variations in hydrophobicity and charge well (Roberts et al., 2015). Interestingly, the ELP structure also provides a sequence context for high readthrough in orthogonal translation, allowing suppression of up to 30 in-frame stop codons in a single polypeptide chain (Amiram et al., 2015). Thus, when position X is chosen as the site of ncAA installation *via* genetic code expansion, ELPs appear to provide an excellent context compared to other protein scaffolds. This is consistent with empirical observations suggesting the existence of so-called mRNA “context effects” in SCS. The insertion of a stop codon into a given genetic sequence can lead to large deviations from natural mRNA folding and subsequently affect the strength of binding site interactions within the ribosome (Gorochowski et al., 2015).

ELPs are a class of self-assembling peptides derived from the water-soluble portion of the tropoelastin protein (Urry, 1997; Vrhovski and Weiss, 1998; Bellingham et al., 2003). Elastin is an extracellular matrix protein found in multicellular organisms as part of connective, skin, lung, and tendon tissues. Due to its high biological similarity to natural tissues, elastin and ELPs are well tolerated and result in a very mild immune response when used for human applications (Hale et al., 2005; Vanderschuren et al., 2022). In addition, ELPs possess a lower critical solution temperature (LCST), which is useful for the development of smart adhesives. The LCST depends on the sequence length of ELPs and the ionic strength of the solution they are located in. Since the length of the ELPs can be precisely genetically encoded, the LCST can be easily adjusted to the desired temperature. A desirable project end goal is to create temperature-controlled ELP-based wet adhesives. By obtaining soluble protein in a biotechnological production setting, purification and application would be facilitated, but upon application to a desired body part (where the temperature is above LCST), the smart adhesive reaches the coacervation point to induce robust adhesion. The combined properties of adhesion (induced by *m*-oNB-Dopa (**1**)) and cohesion (induced by ELP above LCST) are essential for this endeavor.

In this context, we have set the goal to further push the limits of orthogonal translation by rational engineering of the complex system to enable genetic encoding of *m*-oNB-Dopa (**1**) at the scale of up to 60 in-frame stop codons in an ELP gene. To address nitro group reduction *in vivo*, we modified the *Escherichia coli* host by deleting six non-essential nitroreductase genes from the genome. The resulting bacterial strain has drastically reduced nitroreductase activity which enables the intracellular and chemically stable introduction of *m*-oNB-Dopa (**1**) and other nitro group containing ncAAs into peptides and proteins. The overall performance of our combined approach is demonstrated by the production of additional recombinant biomolecules such as the antimicrobial peptide nisin equipped with side chains bearing intact aromatic nitro groups. In this way, we succeeded in establishing a robust, cost-effective and rapid method for the production of nitrated polypeptides through expansion of the genetic code.

## 2 Materials and methods

### 2.1 Plasmid vectors

#### 2.1.1 Orthogonal pairs

All plasmids containing the orthogonal translation system (OTS) were pUltra backbones (Chatterjee et al., 2013). *Mj*ONB-DOPARS and *Mj*ONBYRS were constructed previously (Hauf et al., 2017; Baumann et al., 2019). *Mj*PCNFRS was a gift from Abhishek Chatterjee (Boston College, MA, United States).

#### 2.1.2 Recursive Directional Ligation

Monomer gene fragments encoding the different ELP scaffolds were synthesized by GeneArt (ThermoFisher Scientific, Waltham, MA, United States). The overall cloning strategy is shown in Supplementary Figure S1. To generate the ELP constructs, the monomers were transferred into the high copy pSB1C3 vector (Registry of Standard Biological Parts, 2022). Monomer elongation was performed by digesting the desired plasmid with restriction enzyme BglI followed by dephosphorylation with alkaline phosphatase (FastAP, ThermoFisher Scientific, Waltham, MA, United States). Subsequently, the desired insert fragment was obtained by double digestion with BglI and PflMI and ligation, yielding an elongated ELP scaffold. To verify that the correct insert was formed, an analytical digest of isolated plasmids with XbaI and PstI was performed (*cf.* Supplementary Figure S9). This was necessary because colony PCR with ELP constructs bigger than the ELP(10x amber) resulted in unsuccessful amplifications, likely due to the high GC content and the repetitive nature of the ELP gene. ELP scaffolds >2000 bp (up to the ELP(40x amber) construct) were also analyzed by Sanger sequencing from both directions. When the ELP scaffold reached the desired repeat length, the construct was double digested (with BglI and PflMI) and ligated to a suitable fragment of pET-28a expression vector. Successful DNA assembly was verified by double digestion with BglI and XbaI (Supplementary Figure S9). ELP scaffolds >1,000 bp (up to the ELP(20x amber) construct) were also verified by Sanger sequencing (forward direction).

### 2.2 Analysis of sfGFP expression by intact cell fluorescence

Electrocompetent *E. coli* cells were transformed with the orthogonal translation system and reporter plasmids. LB agar plates contained 1% glucose and corresponding antibiotics. Single colonies were used for inoculation of 2 ml LB (in 14 ml tubes) containing 1% glucose and appropriate antibiotics and grown to saturation overnight. Assays were performed in 96-well plate format. Cultures were added to each well at a dilution of 1:100 in ZYP-5052 auto-induction medium to a final volume of 100 μL supplemented with antibiotics and ncAAs (various concentrations as indicated). Cells were grown in black 96-well flat bottom plates (Greiner Bio-One, Kremsmünster, Austria) covered with a gas permeable foil (Breathe-Easy^®^, Diversified Biotech, Doylestown, PA, United States) and shaken at 37°C for 18 h. For endpoint measurements, the plate foil was removed and fluorescence was measured on a M200 plate reader (Tecan, Männedorf, Switzerland). For OD_600_ measurements, 50 µL of ZYP-5052 medium was added to clear 96-well μ-plates and 50 µL of culture was added. The excitation and emission wavelengths for fluorescence measurements were set to 481 and 511 nm, respectively. Fluorescence values were normalized to the corresponding OD_600_. Biological triplicates (three independent replicates) were used for the measurements of each aaRS construct. Relative fluorescence was normalized to the highest value. The data (incl. Standard deviation) represent the mean of three biological replicates. The experiment which led to Figure 3 was repeated once, with similar results (data not shown).

### 2.3 Recombinant protein expression

For expression of target protein variants, *E. coli* strains were used in 10 ml ZYP-5052 medium supplemented with 1 mM ncAA and appropriate antibiotics. The expression medium was inoculated with a starter culture (1:100). Shake flasks were incubated at 37°C for 20 h while shaking at 200 rpm. Cells were harvested by centrifugation and stored at −80°C or used directly for protein purification.

### 2.4 Recombinant protein purification

Harvested cell pellets were resuspended (50 mM sodium phosphate, 300 mM NaCl, 20 mM imidazole, pH 8.0) and lysed with B-PER Bacterial Protein Extraction Reagent (ThermoFisher Scientific, Waltham, MA, United States) according to protocol, with the addition of phenylmethanesulfonyl fluoride (PMSF, 1 mM final concentration), DNAse and RNAse. The purified lysates were loaded onto an equilibrated Protino Ni-NTA column (Macherey-Nagel, Düren, Germany) and purified using a peristaltic pump (Pharmacia Biotech, now: Cytiva, Marlborough, MA, United States). After washing with 10 column volumes of resuspension buffer, elution buffer (50 mM sodium phosphate, 300 mM NaCl, 500 mM imidazole, pH 8.0) was used to elute the his-tagged target proteins. The first 2 ml (covering the dead volume) were discarded. Subsequently, the eluate (1.5 ml) was collected and dialyzed in cellulose film tubes against 5 L buffer (50 mM sodium phosphate, 300 mM NaCl, pH 8.0) for at least 2 h with three changes into fresh buffer each. The concentrations of purified proteins were determined by measuring the absorbance of the sfGFP chromophore at 488 nm or for amilCP by measuring the absorption at 280 nm.

### 2.5 Decaging of ortho-nitrobenzyl-tyrosine

For decaging of the photocleavable ONBY (**3**) analog in solutions of purified protein or whole cell lysates, samples were placed in a HPLC glass vial. A homemade 365 nm LED lamp (radiant flux ∼720 mW) was used at a distance of ∼3 mm for the indicated time periods.

### 2.6 Time-resolved chromophore maturation of amilCP(Y63ONBY)

Proteins were expressed and purified according to the protocol described above. After purification, 100 µL protein solution at a concentration of 1 mg/ml was transferred to HPLC glass vials (due to strong light absorption, plastic vials were avoided). Irradiation with UV light was performed according to the protocol described above. After irradiation, the samples were transferred to a 96-well clear bottom plate (Greiner Bio-One, Kremsmünster, Austria), and monitored for 24 h. Absorption was measured static at 589 nm using a M200 plate reader (Tecan, Männedorf, Switzerland). This experiment was performed once.

### 2.7 Antimicrobial activity assay

To determine antimicrobial activity of recombinant peptides, an overnight culture of the nisin-sensitive indicator strain *L. lactis* NZ9000 pNZ_nisPT was incubated in M17 medium containing 1% (w/v) glucose (GM17) and 5 μg/ml chloramphenicol at 30°C without agitation (Khusainov et al., 2011; Khusainov and Kuipers, 2013). Fresh GM17 medium was inoculated and cells were grown at 30°C until OD_600_ of 0.4–0.6 was reached. 1 ml of the culture was added to 50 ml of molten GM17-agar supplemented with chloramphenicol and poured into a large Petri dish. Holes were poked into the solidified agar using the wide end of a glass Pasteur pipette (Nickling et al., 2018). As source of the antimicrobial peptides, 1 ml of the *E. coli* expression cultures was centrifuged (3 min, 7,000 *g*) and the cell pellet resuspended in 500 µL buffer (50 mM sodium phosphate, pH 7.4). Cells were lysed by sonification (Sonoplus HD3200, MS72 microtip, Bandelin, Berlin, Germany) at 30% amplitude with pulse cycles of 1 s on and 5 s off for 3 min. Cell debris was removed by centrifugation (4°C, 10 min, 13,000 g). The obtained supernatants were diluted and normalized to 1 ml OD_600_ = 0.6 or 1.0 relative to the initially harvested cell density. 40 µL of each normalized sample was added into the holes of the indicator plate and incubated overnight at 30°C. Corresponding *E. coli* lysates with recombinant nisin containing non-decaged ONBY served as a negative control. Chloramphenicol at a concentration of 400 μg/ml served as a positive control compound. This experiment was performed once.

### 2.8 Intact protein ESI-MS

An Agilent 6530 Q-TOF LC/MS system was used. Samples were infused at a flow rate of 0.3–0.5 ml min^−1^ in a gradient from 5% acetonitrile 0.1% formic acid in water to 80% acetonitrile 0.1% formic acid in water over a Discovery Bio Wide Pore C5 column, 2.1 × 100, 3 micron (Supelco analytical, Sigma-Aldrich, St. Lois, United States) for 20 min. Deconvolution of spectra was performed with Agilent MassHunter Qualitative Analysis software version B.06.00 Bioconfirm Intact Mass Module employing the Maximum Entropy Deconvolution Algorithm. Raw data were plotted using Origin.

### 2.9 Chromosomal gene deletion by homologous recombination

Chromosomal gene deletions were performed according to a modified method of Datsenko and Wanner (Jensen et al., 2015). *Via* PCR, the kanamycin resistance cassette was amplified with the flippase recognition sites (FRT) from genomic DNA of *E. coli* clones obtained from the Keio Single Gene Knockout Collection (Baba et al., 2006). These knockout cassettes can be conveniently PCR-amplified by using either bacterial colonies or bacterial cultures as source of the genomic DNA. Primers were designed to amplify the cassette and, in addition, up to 200 bp of homologous regions up- and downstream of the target genes. Taq polymerase was used since amplification using high-fidelity DNA polymerases (Q5, Phusion) failed. PCR products were purified by gel extraction. Between 100 and 250 ng DNA was used to transform B-95.ΔA cells carrying the pSIJ8 plasmid (Jensen et al., 2015; Mukai et al., 2015). Expression of the pSIJ8-encoded λ-Red recombination system was induced *via* 0.2% arabinose (w/v) at an OD_600_ of 0.3 for 30–45 min during preparation of these electrocompetent cells. After transformation and 2 h recovery (30°C, 220 rpm), cells were plated on LB-Kan agar plates and incubated overnight at 30°C. Homologous recombination of the FRT-*kan*-FRT cassette and the *E. coli* genome was verified by colony PCR. Antibiotic markers were removed from the genome by growing the desired strain at 30°C in LB, inducing the FLP gene with 50 mM L-rhamnose (final concentration) at an OD_600_ between 0.1 and 0.4 for 4–6 h and plating the strain afterwards on LB agar containing 100 µg/mL Ampicillin. Antibiotic resistance cassette removal was again verified by PCR. Then, either the next round of gene deletion was performed as described above, or at the final stage the strain was cured of the pSIJ8 plasmid. The final clone was incubated at 42 °C overnight to cure the strain of the temperature-sensitive pSIJ8 plasmid and verified by PCR. All culturing steps were made in a 14 mL culture tube and 2 mL LB with apropriate antibiotics and additives described above. Loss of the plasmid was confirmed by PCR and by growing corresponding clones on LB agar supplied with and without ampicillin.

## 3 Results and discussion

### 3.1 Optimizing *Mj*ONB-DopaRS activity and fidelity

Tyrosyl-tRNA synthetase from *Methanocaldococcus janaschii* (*Mj*TyrRS) forms the basis for the ribosomal incorporation of all ncAAs used in this study. Previous studies hypothesized that the native enzyme recognizes the *para*-OH group of its natural substrate tyrosine *via* two critical residues, Y32 and D158, in the amino acid binding pocket analogous to *Bacillus stearothermophilus* TyrRS (*Bst*TyrRS). To shift the amino acid substrate recognition from tyrosine to novel ncAA substrates, it was assumed that this natural mode of substrate recognition must be abolished and that D158 is functionally more important than Y32 (Antonczak et al., 2011). In engineered *Mj*TyrRS enzymes, these two residues are usually mutated to smaller residues, as it is the case in the first aaRS reported to activate ONBY (**3**) (Deiters et al., 2006). The same strategy was followed in the design of the *o*NB-Dopa aaRS gene library in our group, with the original goal to genetically encode *p*-*o*NB-Dopa (**2**) (Hauf et al., 2017). Interestingly, the obtained *Mj*TyrRS enzyme recognizes *m*-*o*NB-Dopa (**1**) and not *p*-*o*NB-Dopa (**2**). Therefore, the question arose whether the intentionally fixed mutations Y32A and D158A are indeed required for genetic encoding of *m*-*o*NB-Dopa (**1**) and how they affect the orthogonality and activity of the aaRS. Review of the structural data suggested that there may be an interaction of the Y32 OH group with the *para* OH group of *m*-*o*NB-Dopa (**1**) that could enhance ncAA recognition. Because this had not been explored in the previous work, we reversed these mutations.

**FIGURE 2:**
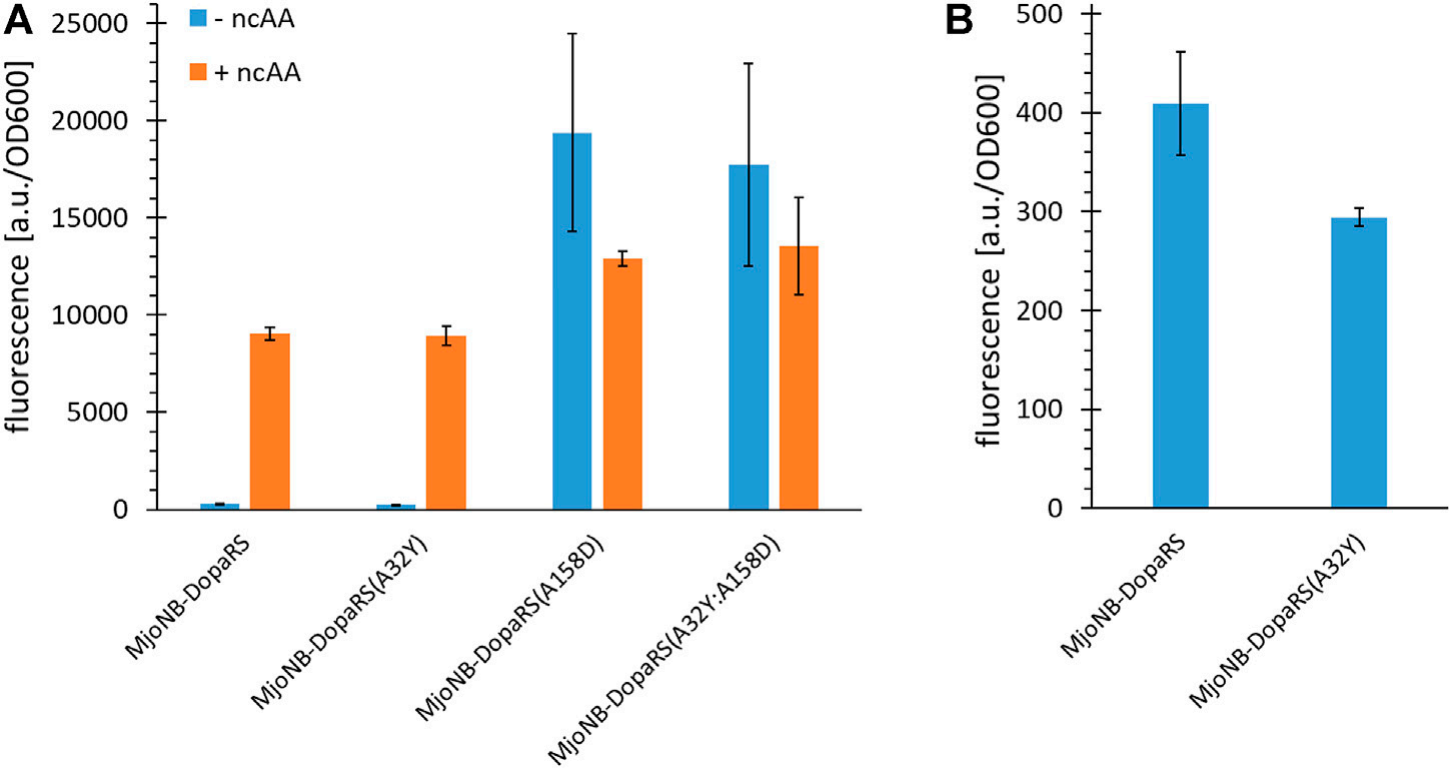
Ribosomal incorporation efficiency of *m*-*o*NB-Dopa upon mutation of the ncAA binding pocket of *Mj*TyrRS. **(A)** OTS efficiency, see main text for a detailed description of the reporter constructs. Ribosomal incorporation with ncAA (+ ncAA = 1 mM *m*-*o*NB-Dopa) and corresponding controls without ncAA supplementation. **(B)** Zoomed in part of (**A**) to visualize differences in background stop codon suppression of the first two setups. Intact cell fluorescence of *E. coli* strain BL21(DE3) with SUMO-sfGFP(R2amber) as reporter protein, endpoint measurements after 18 h of incubation. Fluorescence values were normalized with corresponding OD_600_ values to correct for differences in cell density. Data (incl. Standard deviation) represent the mean of three biological replicates.

This confirmed the hypothesis that residue D158 is more important for Tyr recognition, because the D158A mutation is necessary individually and in combination to maintain aaRS orthogonality, whereas Y32A is not (Figure 2A). Although the A32Y reversion did not improve *m*-*o*NB-Dopa (**1**) incorporation, our results suggest an increased aaRS fidelity, and thus was used throughout this study. The level of background suppression (stop codon readthrough with canonical amino acid incorporation) decreased by around 30% (Figure 2B). This behavior highlights that this amino acid substrate recognition is quite different compared to other *Mj*TyrRS mutants (Dumas et al., 2015; Gueta et al., 2022). Reversion of Y32A generally resulted in complete inactivation of ncAA recognition or a drastic reduction in catalytic activity and fidelity for the corresponding ncAAs (Antonczak et al., 2011). Certain, albeit low, levels of background suppression are often observed for OTSs due to either near cognate suppression or noncomplete orthogonality of aaRS in the absence of the ncAA, which then lead to the incorporation of canonical amino acids (AA). In engineered *Mj*TyrRS variants, tRNA mischarging most likely occurs with Tyr (O’Donoghue et al., 2012; Aerni et al., 2015).

### 3.2 Multisite incorporation of *m*-*o*NB-Dopa into elastin-like-polypeptides at up to 60 positions

**FIGURE 3:**
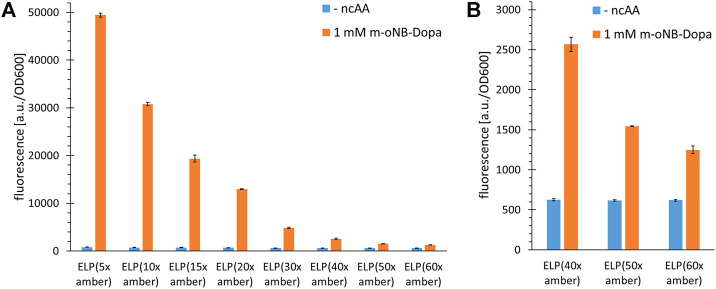
Incorporation efficiency of *m*-*o*NB-Dopa for increasing numbers of installation sites in ELP constructs. Stop codon suppression and OTS constructs as indicated in the main text, see Supplementary Material for sequence details. **(A)** Ribosomal incorporation of *m*-*o*NB-Dopa (1 mM) with ELP constructs containing a varying number (5–60) of in-frame amber stop codons. Controls lack ncAA supplementation (- ncAA). **(B)** Data extract with focus on the suppression of 40, 50 and 60 in-frame stop codons for ncAA incorporation. Intact cell fluorescence of *E. coli* B-95.ΔA (RF1 deficient strain), endpoint measurements after 18 h of incubation. Fluorescence values were normalized with corresponding OD_600_ values to correct for differences in cell density. Data (incl. Standard deviation) represent the mean of three biological replicates.

Approaching more and more ncAA incorporation sites within the repeat-based ELP scaffold required a reliable modular assembly method for the genetic constructs. We decided to prepare them from two monomeric initial sequences using Recursive Directional Ligation (RDL, see Supplementary Figure S1), which is well suited to construct the genes of block/repeat based polymer sequences (Meyer and Chilkoti, 2002). After determining the most efficient *Mj*TyrRS enzyme variant for ribosomal incorporation of *m*-*o*NB-Dopa (**1**), the production of ELP constructs with up to 60 in-frame amber stop codons was attempted (Figure 3). To facilitate readout, ELP variants were fused to the N-terminus of superfolder Green Fluorescent Protein (sfGFP) (see Supplementary Material for sequence information). In this way, translation of the full length ELP gene also leads to translation of sfGFP. As previously noted, translation efficiency can be directly observed with fluorescence measurements (Amiram et al., 2015).

For the efficient incorporation of ncAAs at multiple sites, protein production was performed in a recoded and release factor one (RF1) deficient *E. coli* host derived from BL21(DE3), as in our original study (Mukai et al., 2015; Hauf et al., 2017). In this host (strain B-95.ΔA), the competition between the desired in-frame amber SCS and the endogenous translation termination is abolished. To be consistent with our previous studies and because we did not intend to use the strain in minimal media, we used B-95.ΔA rather than its derivative B-95.ΔAΔfabR. Nevertheless, we would like to note that the latter could improve production in minimal media and at low temperatures (*cf.* (Mukai et al., 2015)). Figure 3 shows that high full length production yields can be achieved for ELP(5x ONB-Dopa). This is consistent with previous reports and also with quantification of small scale protein production yields (for the latter, see paragraph 3.5) (Amiram et al., 2015; Gueta et al., 2022). Reporter fluorescence signals show that constructs with up to 60 stop codons can produce full length protein (Figure 3B). To date, at most 30 stop codons have been suppressed *in vivo* (Amiram et al., 2015; Gueta et al., 2022) and 40 *in vitro*, respectively (Martin et al., 2018). This is intriguing since it is well known that increasing the number of in-frame stop codons normally dramatically decreases the efficiency of translation. We would like to emphasize that the fluorescence signals are an indirect estimate of protein yield. The experimentally determined sfGFP signal depends on a variety of factors including folding efficiency and solubility of the fusion protein. For this screening of increasing incorporation sites, we chose not to include indirect control constructs without stop codons because the physico-chemical properties of natural amino acids (e.g., Tyr, which can be ribosomally incorporated *via* wild-type *Mj*TyrRS) differ significantly from those of ncAAs. While fluorescence-based protein yield estimates for sfGFP constructs with a single site of ncAA incorporation are commonly accurate, fusion protein ELP-sfGFP constructs may behave differently (Martin et al., 2018). It has also been observed that ELP repeat length can correlate with decreasing fluorescence signals, independent of ncAA incorporation efficiency (Amiram et al., 2015; Martin et al., 2018; Hadar et al., 2021). Depending on ELP and ncAA properties, cumulative effects could arise from differences in the strength of gene expression or from perturbations that ELP parts of increasing length exert on sfGFP folding and fusion protein solubility.

Based on our experience with various reporters and MS-based detection of ncAA incorporation into proteins and peptides, we expect a fluorescence signal that is twice as high as the background suppression level to indicate a robust incorporation system. It is worth noting that it remains unknown why such a large number of in-frame stop codons can be suppressed in the ELP scaffold. For other scaffolds such as sfGFP, protein yields decrease dramatically even with only five in-frame stop codons in the gene sequence. To start off with a highly efficient ncAA incorporation system, we initially conducted a prescreening with *Mj*ONBYRS and a second high performing aaRS enzyme with a panel of ncAA substrates (Supplementary Figure S2). This confirmed that the orthogonal translation system (OTS) with *Mj*ONBYRS is highly active and allows the ribosomal incorporation of several ncAAs. Also for O-propargyl- and O-allyl-tyrosine supplied at low ncAA concentrations, it became our preferred aaRS enzyme (Supplementary Figure S4). But even with these efficient OTSs, sfGFP production yields decrease far more upon multisite ncAA installation compared to those of the ELP scaffolds (Supplementary Figure S5). A decrease in translation efficiency of up to 75% was observable when the number of in-frame amber stop codons in sfGFP was increased from one to five. This should be investigated in the future to uncover the underlying higher-level context effects, which could be very useful for orthogonal translation systems in general. It should be noted that the decrease in fluorescence signal could be attributed to misfolding or aggregation of sfGFP caused by incorporation of ncAA, as previously reported for an *in vitro* protein production system (Martin et al., 2018). ELPs are unusual, non-globular polypeptide structures that represent a kind of linear “string” of tandem motifs. We hypothesize that these glycine- and proline-rich repeat structures are predominantly intrinsically disordered. This could facilitate their ribosomal translation, since no fine regulation or balance between translation rate and co-translational folding is required. The result is an excellent context for orthogonal translation with an expanded genetic code.

**FIGURE 4:**
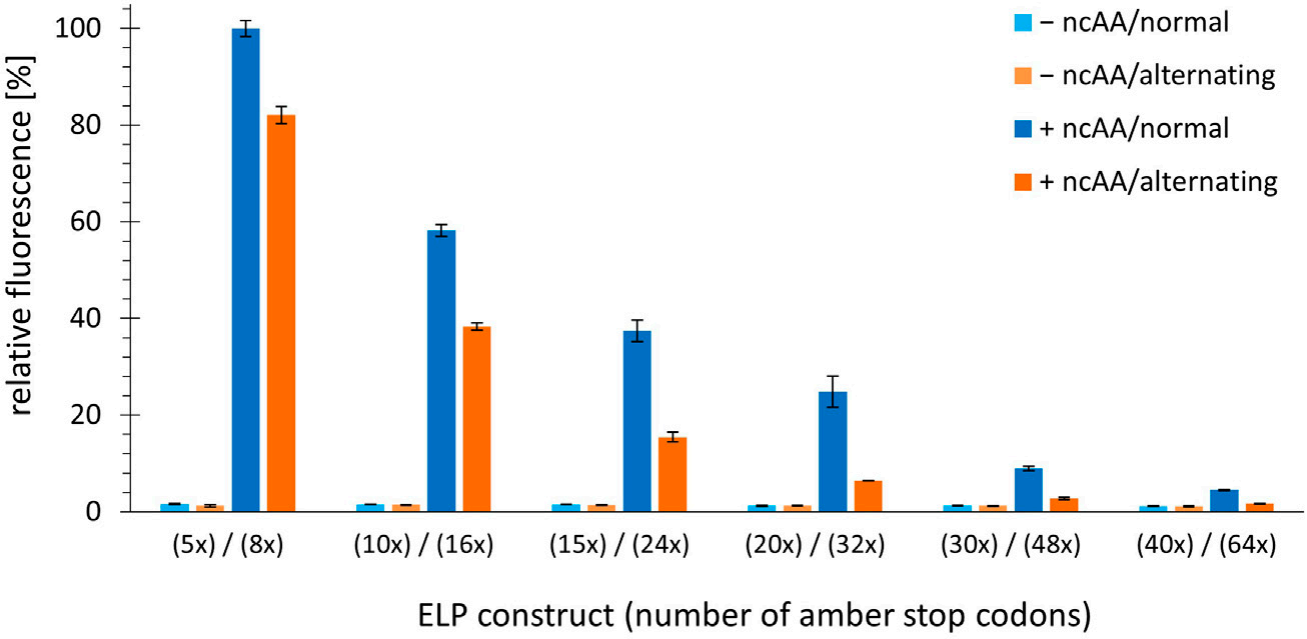
Comparison of OTS efficiency upon alteration of the number and spacing pattern of in-frame stop codons. Ribosomal incorporation of *m*-*o*NB-Dopa (1 mM) into ELP-sfGFP constructs containing a varying number of in-frame amber stop codons. Constructs are shown as pairs of identical ELP fragment length (e.g. ELP(5x amber) and ELP(8x amber)). Normal = one amber stop codon per three VPGXG repeats, equidistant placement. Alternating = alternating amber stop codons, see Supplementary Material for sequence details. Intact cell fluorescence of *E. coli* B-95.ΔA, endpoint measurements after 18 h of incubation. Data (incl. Standard deviation) represent the mean of three biological replicates.

The fact that the fluorescence signal decreases in response to increasing the number of stop codons (most likely because truncation products are formed) despite the absence of endogenous RF1 suggests that the OTS pair cannot deliver enough charged tRNA to prevent ribosome stalling. For a given translation rate, increasing the distance between stop codons could provide more time for loading and delivery of the orthogonal tRNA, which in turn could lead to improved protein production. To better understand the correlation between the number and spacing of in-frame stop codons within the ELP sequence, constructs with alternating stop codon replacements were used, combined with the same fluorescence readout as before. Notably, we cloned and produced construct pairs with identical ELP length to control for the potential impact on the fluorescence reporter. ELP constructs with multiples of five stop codons were built using ELP(5x amber) as origin, while those of with multiples of eight where built using the ELP(8x amber) construct. Details of these constructs can be found in Supplementary Table S1. Using these pairwise comparisons, we found that the amount of full-length protein produced was predominantly correlated with the total number of in-frame stop codons; the distance between these and the overall ELP length had little to no effect (Figure 4). This is best observed by comparing equidistant ELP constructs with their counterparts showing alternating placement of amber codon (e.g. ELP(10x amber) vs ELP(16x amber)). Increasing the number of stop codons and ncAA incorporation sites by 60% leads to a corresponding decrease in the fluorescence signal. Even when the ELP repeat length and the number of stop codons reach high values (and the overall reporter signals are low), the same picture emerges. This can be seen, for example, when comparing the results of ELP(30x amber) (normal, equidistant placement) with ELP(32x amber) (alternating stop codon placement). Although the ELP part of the ELP(30x amber) construct is much larger (495 vs 335 AA), both constructs appear to be produced at comparable levels, suggesting that stop-codon readthrough is the most important determinant. As mentioned earlier, longer ELP length may also contribute to a reduction in the reporter fluorescence signal. However, this effect seems to be less pronounced in our pairwise comparison, e.g. for ELP(15x amber, normal) and ELP(16x amber, alternating). These differ only by one ncAA incorporation site but significantly in ELP fragment length (255 vs 175 AA). A comparable full-length reporter signal was observed, differences cannot be resolved. The high proportion of hydrophobic residues in the ELP part could increase the tendency of the fusion protein to aggregate once the ELP portion reaches a substantial length. At the ELP(15x amber) and ELP(24x amber) construct pair stage, the ELP and sfGFP parts are approximately equal in length. To sum up, constructs with a similar total number of stop codons result in a similar fluorescence signal in the cell, suggesting a comparable yield of full-length protein. To better understand this argument, we visualized the data from Figure 4 differently by omitting the columns without ncAA and sorting the constructs by the number of in-frame stop codons. In the resulting Supplementary Figure S3, we see that the reporter signal intensity follows the number of in-frame stop codons. Returning to the initial hypothesis: Increasing the distance between or changing the pattern of in-frame stop codons by the measures used herein does not facilitate ribosomal readthrough.

### 3.3 Analysis of recombinant ELP-sfGFP fusion protein

**FIGURE 5:**
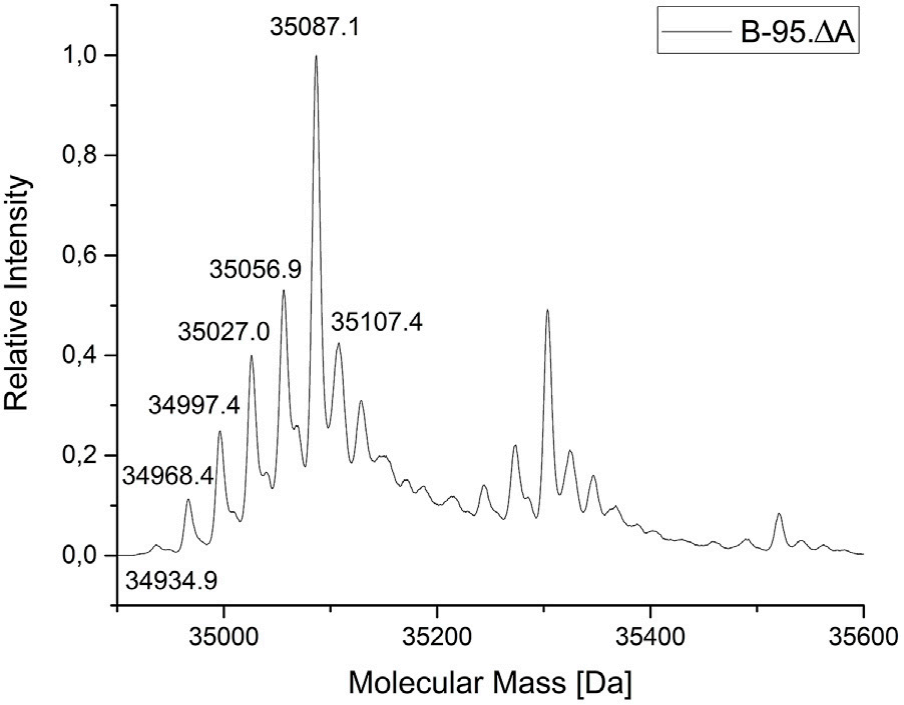
Mass profile (ESI-MS) of ELP(5x *m*-*o*NB-Dopa)-sfGFP produced in *E. coli* B-95.ΔA. The peak pattern shows a characteristic mass shift in line with nitro group reductions (with mass differences fitting to theoretical values for the reduction of the ncAA nitro group). Deconvoluted intact protein mass values and shifts are given in Table 1. The main peak (35087.1 Da) indicates the target species with five ncAAs and devoid of nitro group reduction. The corresponding chemistry is explained in the main text.

For a more detailed analysis of the protein species produced, the ELP(5x ONB-Dopa)-sfGFP construct was prepared with five ncAA incorporation sites at the scale of 50 ml shake flask cultures. The purified target protein samples were subjected to ESI-MS analysis. Figure 5 shows the successful incorporation of five *m*-*o*NB-Dopa (**1**) moieties into the full-length protein. Unfortunately, but not unexpectedly, a significant portion of the protein produced (approximately 50% of the total as estimated from peak intensities) contains one to five reduced nitro groups. For clarity, the measured mass shifts of the corresponding protein species in Figure 5 are shown and interpreted in Table 2. It can be seen that the observed intact protein masses agree very well with those predicted for possible nitroreduction species. The differences between the expected and observed masses become larger for the less abundant protein species as the measurement confidence is correlated. Attempts were made to analyze constructs containing more than five *m*-*o*NB-Dopa (**1**) moieties, but because of the increasing size of the ELP part, only insoluble fusion proteins were obtained. These were not compatible with the established ESI-MS analysis protocol. Methods exist to analyze such constructs, for example, by proteolytic digestion, but further efforts were discontinued once we found better production methods for proteins with nitro group containing ncAAs, as described below (Israeli et al., 2021; Vanderschuren et al., 2022).

**TABLE 1:** ESI-MS analytics of ELP(5x *m*-*o*NB-Dopa)-sfGFP produced in *E. coli* B-95.ΔA.

Species^a^	Calculated mass [Da]	Found mass [Da]	Δ mass [Da]
mat., unreduced	35087.1	35087.1	0
mat., 1x reduced	35057.1	35056.9	0.2
mat., 2x reduced	35027.1	35027.0	0.1
mat., 3x reduced	34997.1	34997.4	0.3
mat., 4x reduced	34967.1	34968.4	1.3
mat., 5x reduced	34937.1	34934.9	2.2
Non-maturated, unreduced	35107.1	35107.4	0.3

a All protein species without the starting Met, mat. = sfGFP fluorophore is maturated.

**TABLE 2:** *E. coli* genes with known nitroreductase activity which were targeted for deletion.

Gene	Description	Nitroreductase activity^a^	References
*nfsA*	Major oxygen-insensitive nitroreductase	High	Zenno et al. (1996a), Copp et al. (2017)
*nfsB*	Minor oxygen-insensitive nitroreductase	Middle	Zenno et al. (1996b), Copp et al. (2017)
*azoR*	FMN dependent NADH:quinone oxidoreductase (NADH-azoreductase) which can reduce azo dyes. Nitroreductase activity was observed with ortholog from *Rhodobacter sphaeroides*	Middle/Low	Mercier et al. (2013), Copp et al. (2017)
*ydja*	One of the smallest nitroreductases from *E. coli*	Low	Choi et al. (2007), Copp et al. (2017)
*nemA*	Flavin-dependent oxidoreductases related to the old yellow enzyme family	Low	Williams et al. (2004), Copp et al. (2017)
*rutE*	Conserved nitroreductase domain type. Required for growth on uridine. Reduces malonic semialdehyde to 3-hydroxypropionic acid	Low	Kim et al. (2010), Copp et al. (2017), Loh et al. (2006)

a Estimation based on the reported activities.

### 3.4 Engineering a bacterial strain with reduced nitroreductase activity

As initially outlined, bacterial host cells generally reduce aromatic nitro groups in peptides and proteins (Nguyen et al., 2014; Ren et al., 2015; Baumann et al., 2019; Böcker et al., 2019). For the ncAAs employed in this work, the nitro group of moiety **1** is reduced to the corresponding amine *in vivo*. This eliminates the photocleavable ability of the protecting group and ultimately the controlled generation of Dopa-containing recombinant proteins. After reviewing the genome sequence and prevalent literature, at least 11 *E. coli* wild-type genes can be linked to nitroreductase activity (Mercier et al., 2013; Copp et al., 2017). In particular, the NAD(P)H-dependent nitroreductases NfsA and NfsB have the highest ability to reduce nitro groups, e.g. with nitrofurazone (Zenno et al., 1996a; Valle et al., 2012; Copp et al., 2017). Unfortunately, deletion of *nfsA* and *nfsB* has been shown to be insufficient to prevent nitro group reduction for ncAAs in *E. coli* (Ren et al., 2015), even though biotransformation of certain other small molecules can be prevented by only these two deletions (Valle et al., 2012). Following this train of thought, we set out to inactivate six *E. coli* enzymes known to exert nitroreductase activity (Table 2). Gene knockouts were performed in the *E. coli* B-95.ΔA strain as a robust and efficient production platform for the target proteins, as seen above (Mukai et al., 2015).

**FIGURE 6:**
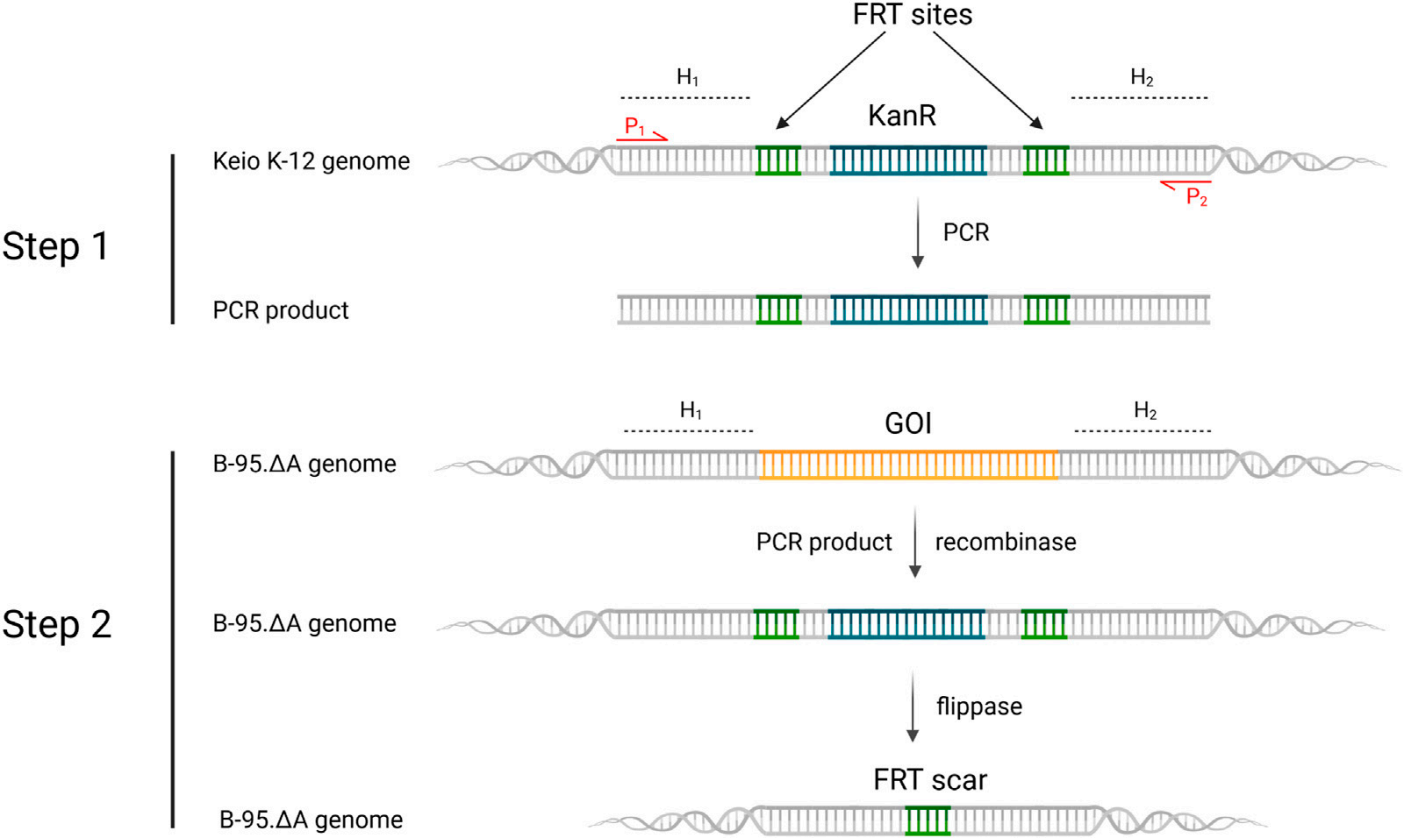
Revised lambda red recombineering workflow for gene deletions leading to the generation of the sextuple *E. coli* knockout strain B-95.ΔA NK53. H_1,2_ = homology region 1 or 2; P_1,2_ = Primer 1 or 2; KanR = kanamycin resistance cassette; GOI = gene of interest, which is targeted for deletion. Step 1 displays the PCR-amplification of the FRT-KanR-FRT-cassette from the Keio knockout strain. Step 2 depicts the recombinase induced replacement of the gene of interest with the FRT-KanR-FRT-cassette and its subsequent flippase catalyzed removal. Created with BioRender.com.

For such a high number of targets, gene deletions were performed with a revised Lambda Red recombineering system (Jensen et al., 2015) using a single plasmid version of the established method of Datsenko and Wanner (Datsenko and Wanner, 2000). In contrast to the latter, the antibiotic resistance cassette with homologous DNA flanks was amplified from the genomic DNA of *E. coli* clones from the Keio Single Gene Knockout Collection rather than from a template plasmid (Baba et al., 2006). Since these single knockout clones contain the antibiotic resistance cassette with flanking FRT sites, the procedures of PCR amplification, knockout in the target strain and cassette excision are compatible. The workflow is shown in Figure 6 and has been described previously in different variants (Meuskens et al., 2017; Swings et al., 2018; Aedo et al., 2019; Rodríguez-Rojas et al., 2020). This hybrid approach has the advantage that the primer binding sites and the length of the homology regions (surrounding the targeted gene) can be chosen freely. It eliminates primer length constraints and helps to avoid bad primer metrics that otherwise compromise primer synthesis and PCR efficiency. Furthermore and notably, this benefits the recombineering efficiency, which is correlated to the length of the homology region (Murphy, 1998; Liu et al., 2003; Sharan et al., 2009). For one or a few deletions, sufficient knockout efficiencies can be achieved with the standard homology region length of 35–50 bp. However, for each deleted gene, an FRT scar remains in the genome after cassette excision. This increases the likelihood that the next antibiotic cassette will be inserted at this scar site instead of the target gene, resulting in false positive knockout clones. Longer homology regions alleviate this effect. Another advantage is that the Keio knockouts were carefully designed to reduce negative effects that can occur when a deleted gene is part of an operon for example (Yamamoto et al., 2009). Homology lengths of 100–200 bp were chosen for our nitroreductase knockouts, which are easily and cost-effectively obtained using desalted primers. After each round of deletion, clones were probed *via* colony PCR and gel electrophoresis to verify target gene replacement and cassette excision, respectively. Sanger sequencing of the genomic PCR products was also performed.

**FIGURE 7:**
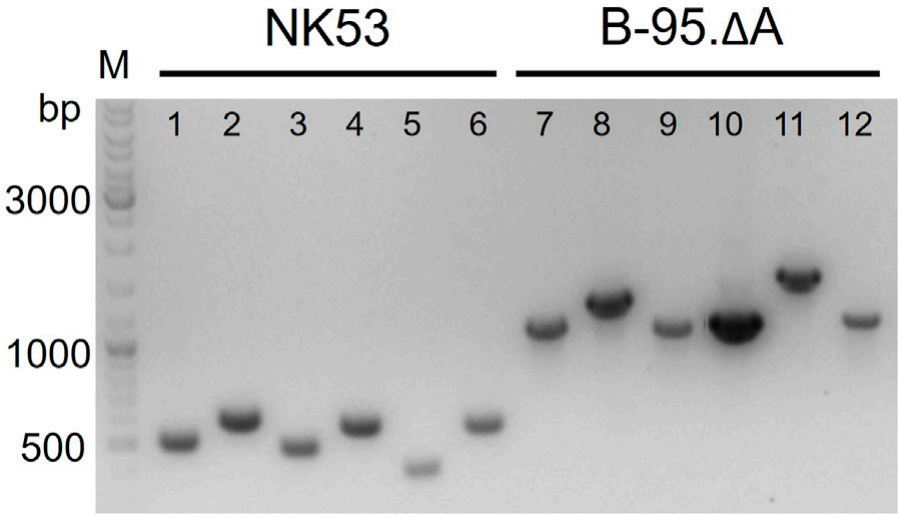
Verification of gene deletions by agarose gel electrophoresis of genomic PCR products. Smaller PCR product sizes indicate deletions within the amplified DNA region. Comparison of knockout strain B-95.ΔA NK53 to its progenitor *E. coli* B-95.ΔA used as source of genomic DNA. Lanes: 1) ΔnfsA:FRT; 2) ΔnfsB:FRT; 3) ΔazoR:FRT; 4) Δydja:FRT; 5) ΔnemA:FRT; 6) ΔrutE:FRT; lanes 7–12 are the corresponding wild-type equivalents.

Consecutive knockouts of the six genes from Table 2 yielded strain B-95.ΔA (ΔnfsA:FRT, ΔnfsB:FRT, ΔazoR:FRT, Δydja:FRT, ΔnemA:FRT, ΔrutE:FRT), herein referred to as B-95.ΔA NK53. This final strain was rescreened for all intended deletions, with the original strain serving as negative control (Figure 7, further exemplified for the *nfsB* deletion in Supplementary Figure S6). Figure 7 clearly shows that the genomic DNA amplification PCR products for all targeted genes are shorter than the equivalents of the original strain, indicating successful deletion of the genes. After six gene deletions, the potential impact on the fitness of the bacterial strain was evaluated by parallel growth assays in 24-well plates, monitoring OD_600_ as a proxy for cell density. To sample a variety of growth environments, strains B-95.ΔA, B-95.ΔA NK53 and BL21(DE3) were each grown in complex rich medium (LB, DYT), complex rich buffered medium (TB, ZYP5052) and chemically defined new minimal medium (NMM) without amino acid supplementation (Budisa et al., 1995; Studier, 2005). Interestingly, B-95.ΔA exhibited enhanced growth and also reached higher final densities than the other two strains. Strain B-95.ΔA NK53 was always on par with BL21(DE3), suggesting that the gene knockouts caused slight fitness deficits compared to the parent strain. But as mentioned earlier, the overall fitness still remains high on the level of BL21(DE3), which is the widely used gold standard strain for high-level protein production (Supplementary Figure S7, S8).

The strategy used herein to generate bacterial host strains with multiple gene knockouts turned out to be very efficient in practice. The generation of longer homology arms in the linear DNA fragment increased the efficiency for multiple knockouts in the same strain. Experience in our laboratory had shown that off-target integrations occur frequently when three or more FRT scars are present in the genome. For each targeted gene knockout by deletion, the selection and verification of only five clones was always sufficient to find a clone with the intended modification. It should be mentioned that there are a variety of genomic engineering strategies, including several scarless alternatives (Fels et al., 2020).

### 3.5 Elucidating the reduced nitroreduction by strain B-95.ΔA NK53

**FIGURE 8:**
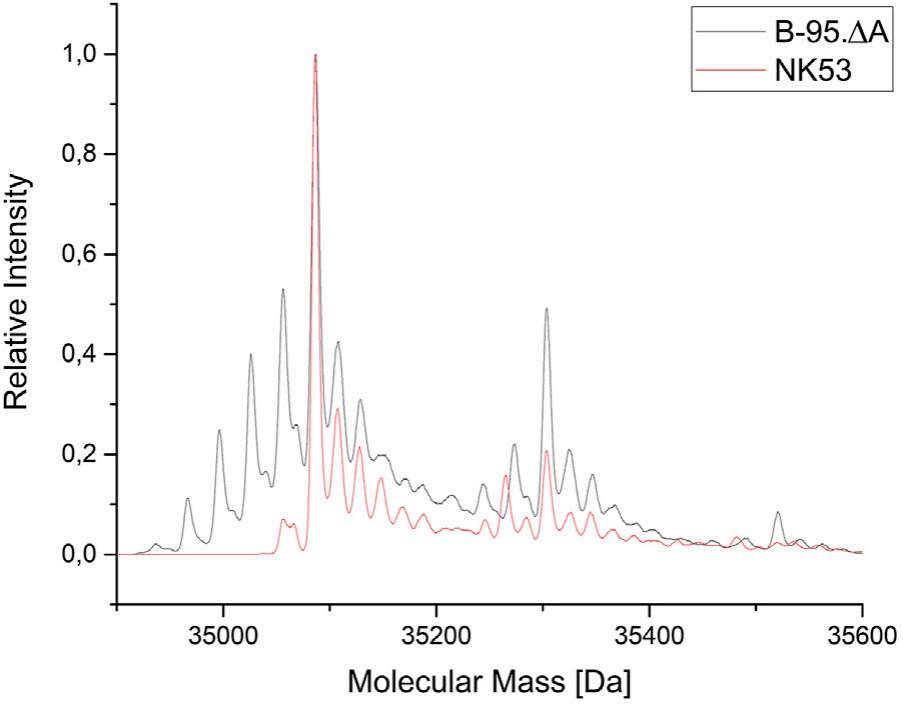
Mass spectrometric profile (ESI-MS deconvolution) of proteins produced in the *E. coli* B-95.ΔA NK53 strain with attenuated nitroreductase activity compared to its progenitor strain. ELP(5x *m*-*o*NB-Dopa)-sfGFP produced by progenitor strain B-95.ΔA (grey) indicates the reduction of several ncAA nitro groups. See Section 3.3 for detailed peak annotation of reduced protein species. Expected intact protein mass for five intact *m*-*o*NB-Dopa moieties = 35087.1 Da. Observed mass: 35086.9 Da. Expected mass upon nitro group reduction at one site: 35057.1 Da. Observed mass: 35057.5 Da.

To evaluate the benefits of the performed gene deletions, the same reporter protein construct as in Figure 5 (ELP(5x *m*-oNB-Dopa)-sfGFP) was produced in the constructed sextuple knockout strain B-95.ΔA NK53, purified, and analyzed by ESI-MS. Protein yields ranged between 60–80 mg/L of culture for both strains. Superposition of Figure 5 and the MS spectrum for B-95.ΔA NK53 purified reporter resulted in Figure 8. Here, it is clear that almost no target protein suffering from nitro group reduction is present when produced by the knockout strain. Only a small fraction of target protein containing a reduced nitro group is detectable, rendering the genome engineering efforts of B-95.ΔA a success. Judging from the spectral peaks (which only provide a rough quantification), about 3% of the total protein contains one reduced nitro group. For this protein which contains five *m*-oNB-Dopa incorporation sites, each of which is susceptible to the undesired PTM, this corresponds to an overall reduction of 94% compared to the progenitor strain.

#### 3.5.1 Applications of nitrated proteins and peptides

**FIGURE 9:**
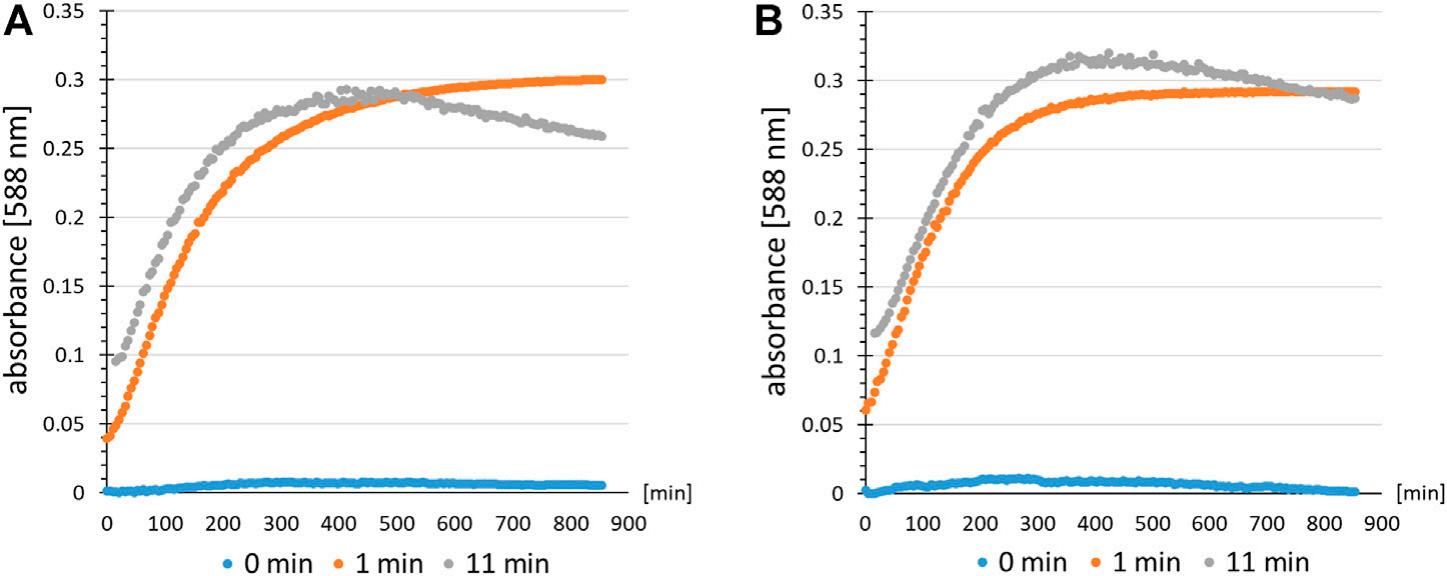
Time resolved chromophore maturation of amilCP(Y63ONBY) detected by an absorption assay after light-induced ncAA decaging. Colored dots indicate the time for which the protein solution of amilCP(Y63ONBY) was irradiated with UV-light prior to data recording (absorbance at λ = 588 nm). Controls (0 min, blue) are without irradiation. Comparison of proteins produced using *E. coli* strain **(A)** B-95.ΔA NK53 or **(B)** B-95.ΔA.

Next, the practical applicability of the B-95.ΔA NK53 strain for the incorporation of other nitro group-containing ncAAs was evaluated. Two types of experiments were conducted. First, we investigated whether differences in nitroreduction of another ncAA, ortho-nitrobenzyl-tyrosine (ONBY (**3**)), could be detected depending on the production strain. The orthogonal translation system consisting of tRNA and evolved aaRS for the incorporation of ONBY (**3**) has been described previously (Baumann et al., 2019). In the first experiment, ONBY (**3**) was incorporated at position Y63 (corresponding to the chromophore position Y66 in GFP) of amilCP, a chromoprotein from the coral *Acropora millepora* (Alieva et al., 2008). Photo-induced cleavage of the nitrobenzyl group should restore the caged Tyr and reinstate the maturation ability of the chromophore. It should therefore allow spatiotemporal control of chromophore maturation. Previously, a similar experiment was performed with a GFP derivative (Groff et al., 2010). Since amilCP has a similar tertiary protein structure, it was assumed that light-induced chromophore maturation would also be possible. The target protein amilCP(Y63ONBY) was produced both in B-95.ΔA and in the engineered B-95.ΔA NK53 strain. Purified proteins were normalized to the same concentration and irradiated with UV-light to deprotect ONBY (**3**) (Figure 9). Absorbance at 588 nm was monitored as a function of time as an indicator of amilCP chromophore maturation. As hypothesized, both protein batches allowed the photoinduced deprotection of ONBY (**3**), leading to autocatalytic chromophore formation of amilCP. To our surprise, no profound differences in chromoprotein absorption were detectable between proteins produced in B-95.ΔA or B-95.ΔA NK53, respectively. If there would have been a difference in the amount of reduced nitro groups of ONBY (**3**) depending on the expression strain, the observed chromophore absorption should have been significantly lower for amilCP(Y63ONBY) produced in the B-95.ΔA strain. We conclude that the reduction of ONBY (**3**) must occur after its ribosomal incorporation into the polypeptide chain, implying that the aaRS can discriminate between ONBY (**3**) and the corresponding reduced substrate (Baumann et al., 2019). It is plausible that the β-barrel structure of amilCP surrounding the chromophore motif can shield ONBY (**3**) from the enzymes present in the cytoplasm of the host that catalyze nitroreductions. This constellation may explain why reduction of ONBY (**3**) also failed to occur in the earlier GFP work (Groff et al., 2010). Confirming this hypothesis, ESI-MS measurements of proteins produced in both strains revealed that no detectable nitroreduction had occurred (Supplementary Figure S10, S11). Consequently, protection by the protein scaffold allows efficient production of ONBY-containing chromoproteins in both strains and nitroreductase knockouts do not confer any benefits.

**FIGURE 10:**
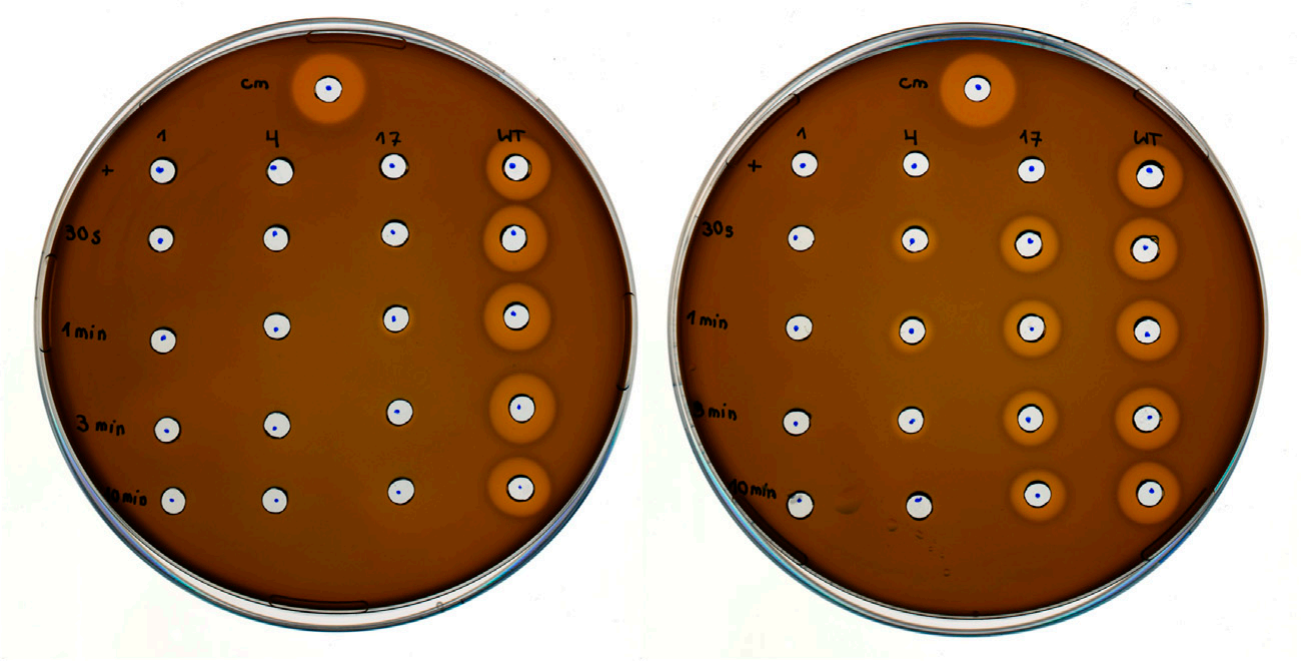
Antimicrobial activity of photocaged and decaged (UV-irradiated) nisin derivatives. *L. lactis* indicator strain assay using *E. coli* cell lysates containing nisin modified with ONBY (3) at position I1, I4 and M17, respectively, with recombinant wild-type nisin (WT) as reference. *E. coli* lysates were prepared with either B-95.ΔA (progenitor, left) or B-95.ΔA NK53 (nitroreductase knockout, right) as host strain. Cm = Chloramphenicol (400 μg/ml final) positive control, first row (+) samples were likewise prepared with ONBY (3) supplementation, but without UV-light irradiation of the *E. coli* cell lysate. Numbers from top to bottom indicate the duration of UV-light irradiation (30 s, 1, 3, 10 min). Numbers above the column indicate the position within the nisin peptide where ONBY (3) is introduced by amber suppression and genetic encoding of the ncAA.

The next recombinant target was the post-translationally modified antimicrobial peptide nisin. This lanthionine-containing peptide antibiotic (lantibiotic) is naturally produced by *Lactococcus lactis* (*L. lactis*). Indicator strain assays can be performed as a readout of the antimicrobial activity of this ribosomally synthesized and post-translationally modified peptide (RiPP; produced by natural hosts or recombinant systems) (Baumann et al., 2017; Nickling et al., 2018). Halos indicate growth inhibition zones on agar plates where the nisin-sensitive *L. lactis* indicator strain cannot grow. Here, we attempted to produce nitro group containing nisin variants in which ONBY (**3**) was incorporated at permissive sites. Due to the complex pathway of ribosomal precursor production, posttranslational modification by NisBC and NisP-catalyzed prepeptide cleavage, the contributions of production levels and specific antimicrobial potency to the observed activities are not easily dissected. Nevertheless, we set out to test whether photoactivatable nisin variants could be produced. By combining the recombinant nisin production system and the orthogonal translation system, positions I1, I4 and M17 of nisin were individually replaced with ONBY (**3**). A previous study by the Kuipers lab showed that these positions can be replaced by Trp and that the corresponding peptides continue to be cleaved by NisP (Zhou et al., 2016). Due to the presence of the photocleavable protection group, we anticipated a change in antimicrobial activity upon UV irradiation. Depending on the amount of reduced ONBY nitro groups, the ability to photocleave should be affected. Consequently, the activity of nisin variants with a high degree of nitroreduction should be less affected by irradiation. As shown in Figure 10, only the recombinant nisin produced in the nitroreductase-deficient strain showed a dramatic increase in antimicrobial activity upon UV-irradiation, which is associated with ONBY (**3**) decaging. Production in the progenitor strain B-95.ΔA revealed a low level of photoactivation of nisin (M17ONBY) and all other constructs yielded inactive preparations regardless of irradiation time. Therefore, only recombinant production of ONBY (**3**) modified nisin from the nitroreductase knockout strain will be discussed further. By far the strongest increase in activity upon irradiation is observed when ONBY (**3**) is introduced at nisin position 17, which is a Met residue in the native peptide and part of ring C (see (Rink et al., 2007) for an illustration of nisin structure and numbering). Mutational studies have shown that modulation of this ring leads to major changes in antimicrobial properties (van Kraaij et al., 2000). The physico-chemical properties of ring C amino acids have a major impact on the antimicrobial activity, which requires precise orientation to and interaction with the target cell membrane (Breukink et al., 1998). The corresponding side chain modification on nisin residue I4 resulted in moderate activity after radiation. Even before irradiation, a small halo was detected, indicating activity. Position 4 is part of nisin ring A and tolerates replacement by Phe without complete loss of activity (Rink et al., 2007). Nevertheless, the exchange of I4 with the ncAA moiety appears to be structurally too different to generate a highly active peptide or sufficient quantities thereof, respectively. When ONBY (**3**) was incorporated at position I1, no antimicrobial activity was observed regardless of irradiation, although a previous study showed that I1Y and I1W mutants of nisin can retain some activity (Lagedroste et al., 2019). This could be due to inefficient production, differences in assay sensitivity, or the fact that the nisin N-terminus is inserted into the target membrane surface, which requires a specific range of side chain hydrophobicity and size. We would like to note that the nisin experiment described above was intended as a snapshot for the applicability of the nitroreductase knockout strain. Because the purification and molecular analysis of ncAA-modified nisin peptides is challenging, the results obtained must be treated with caution. However, because the observed effects were large and consistent with our hypothesis and the data accumulated in this study, we felt confident to include these results.

This experiment demonstrates that, particularly for peptides lacking a tertiary structure, the bioactive function can be efficiently masked (and subsequently activated) when expressed in strains that can protect the *o*NB group from reduction. The solvent accessibility of the ncAA side chain increases both the chance for the reduction of its nitro group by endogenous nitroreductases and the change in physico-chemical properties upon photocleavage. Our examples of ncAA-modified ELPs and a light-activatable lantibiotic show that in particular peptide-based biomolecules can benefit greatly from having a nitroreductase deficient *E. coli* strain available for recombinant production.

## 4 Conclusion

By merging three bioengineering streams (orthogonal translation system, protein scaffold and *E. coli* strain), we have created a platform for the efficient production of polypeptides containing multiple installations of nitro group containing non-canonical amino acids. Fluorescence assays show that ELPs containing *m*-oNB-Dopa (**1**) at up to 60 sites can be produced with as little as 1 mM ncAA supplied. The generated host strain with reduced nitroreductase activity has great potential for the general production of peptides and proteins containing non-reduced nitro groups, even beyond *m*-oNB-Dopa (**1**) and ONBY (**3**). Likewise, it can be used for cell-free protein synthesis, as reported for its progenitor strain (Seki et al., 2018). The strategy described herein can also serve as a template for dramatically reducing the nitroreductase activity of other *E. coli* laboratory strains. Sextuple knockouts can be generated within 1 month using standard lab equipment and consumables. While the genes chosen for knockout significantly reduce the endogenous reduction of nitro groups of solvent-exposed side chains of peptides and proteins, other nonessential genes with this activity remain. These can now be targeted to further reduce unwanted side chain conversions and to further increase the *in vivo* half-life of intact nitro groups. All types of applications where nitro groups in biomolecules are important for protection or mechanistic function can benefit from our engineered strain and approach. By using a robust RF1 knockout strain in combination with an efficient orthogonal translation system, we achieved a high level of amber suppression in ELP genes, resulting in satisfactory amounts of target protein. We envision that the high protein yield combined with the photocleavability of *m*-oNB-Dopa (**1**) and the thermo-responsiveness of ELPs will lead to promising spatio-temporal and temperature controlled smart materials.

## Data Availability

The original contributions presented in the study are included in the article and Supplementary Material. Further inquiries can be directed to the corresponding author.
