# Supplementary material for: Engineered bacterial host for genetic encoding of physiologically stable protein nitration
Source: Front Mol Biosci. 2022 Oct 24;9:992748. doi: 10.3389/fmolb.2022.992748 (PMC9638147; doi:10.3389/fmolb.2022.992748)
Supplement: Supplementary file 1 [file DataSheet1.docx]

Supplementary Material

Engineered Bacterial Host for Genetic Encoding of Physiologically Stable Protein Nitration

Nikolaj G. Koch^1,2^, Tobias Baumann^2^, Jessica H. Nickling^2^, Anna Dziegielewski^2^, Nediljko Budisa^2,3*^

^1^Bioanalytics Group, Institute of Biotechnology, Technische Universität Berlin, Berlin, Germany

^2^Biocatalysis Group, Institute of Chemistry, Technische Universität Berlin, Berlin, Germany

^3^Chemical Synthetic Biology Group, Department of Chemistry, University of Manitoba, Winnipeg, MB, Canada

**Table of Contents**

[1 Supplementary Figures and Tables 3](#_Toc113447746)

[1.1 Supplementary Figures 3](#_Toc113447747)

[1.2 ESI-MS Spectra 12](#_Toc113447748)

[1.3 DNA Sequences and Details of ELP-sfGFP Constructs 14](#_Toc113447749)

[2 Supplementary References 28](#_Toc113447750)

# Supplementary Figures and Tables

## Supplementary Figures

Bacterial expression vectors containing ELP repeat sequences of various lengths were generated using the following approach. See Section 1.3 for sequence information.


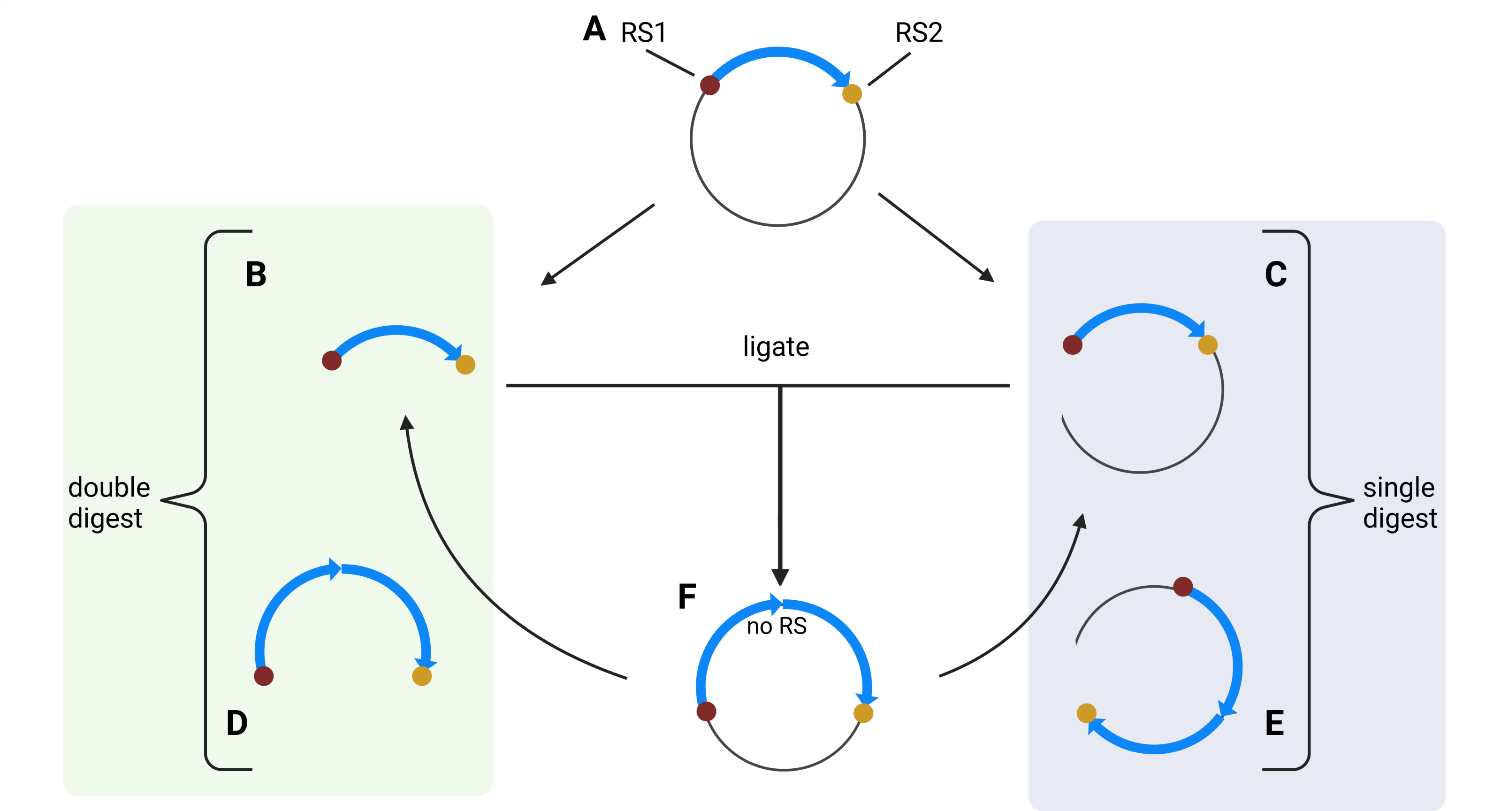


Figure S1. Flowchart of ELP construct design and strategy for genetic assembly using recursive directional ligation (RDL) (Meyer and Chilkoti, 2002). The restriction sites and corresponding enzymes (RS) are: RS1 = PflMI (dark red dot), RS2 = BglI (yellow dot). DNA digestion yields fragments with compatible cohesive (“sticky”) overhangs. A) initial construct, B) ELP gene fragment (blue), C) digested backbone fragment (digested with RS1), D) elongated ELP gene fragment, E) digested backbone fragment (digested with RS2) containing an elongated ELP gene. A single digest (blue shading) is required to generate a backbone fragment suitable for insertion of a desired ELP fragment. A double digest (green shading) is used to generate an ELP gene fragment that can subsequently be used to elongate a desired ELP gene. Importantly, the internal ligation junction in the circular plasmid product F) does not harbor the sequence of RS1 or RS2, respectively.

Example:

Suppose there is a plasmid DNA vector **A** in which an ELP gene is present. To elongate this ELP gene, one needs to digest **A** with RS1 and RS2 and then separate the resulting ELP gene fragment from the vector backbone (**A**→**B**). The recipient vector is created by cutting **A** with just one restriction enzyme (RS1 or RS2, **A**→**C**). Finally, to elongate the target ELP gene, one has to join **B** and **C** by ligation of these two DNA fragments. This creates the circular vector containing the new elongated gene, **F**. Using the same procedure, this vector can be used again to extend the ELP gene.

To gain insights into the efficiency of *Mj*ONB-DopaRS and a second aaRS variant, both of which incorporate ncAA into a different protein scaffold (sfGFP) than ELP, a prescreening was performed using *Mj*ONBYRS (Baumann et al., 2019) and *Mj*PCNFRS (Schultz et al., 2006; Young et al., 2011) with several ncAAs (Figure S2).


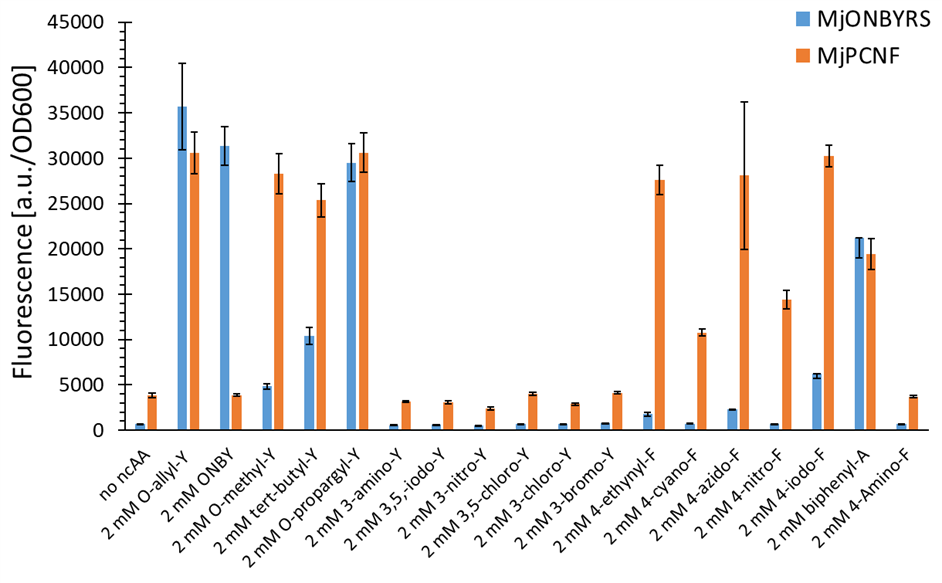


Figure S2. Prescreening of several ncAAs with *Mj*ONBYRS and *Mj*PCNFRS for ribosomal incorporation into sfGFP. Measured fluorescence intensity of intact *E. coli* BL21(DE3) cells expressing the SUMO-sfGFP(R2 amber) reporter and co-expressing the OTS. Endpoint measurements after 24 h with 2 mM ncAAs supplied. Data (incl. standard deviation) represent the mean of three biological replicates.


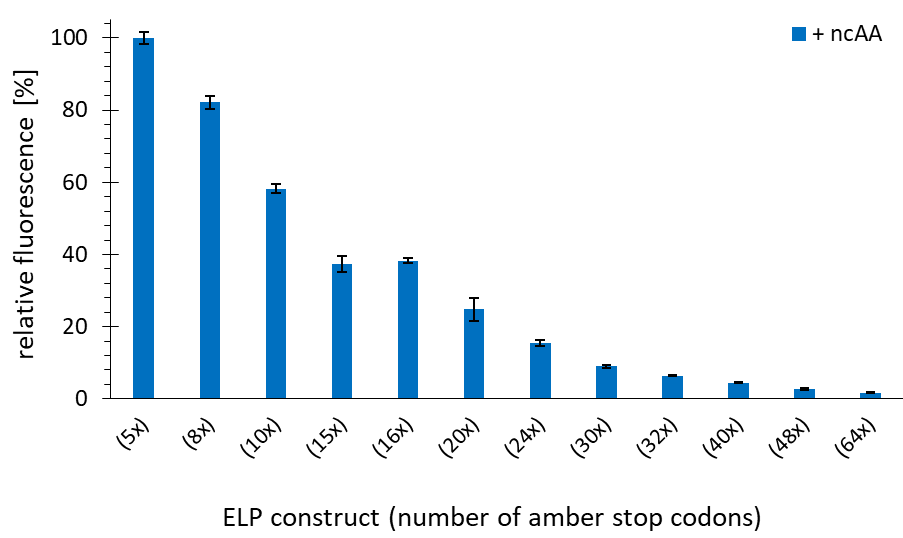


Figure S3. Comparison of OTS efficiencies. The columns are sorted based on the number of in-frame stop codons. Ribosomal incorporation of *m*-*o*NBDopa (1) (1 mM) into ELP-sfGFP constructs containing a varying number of in-frame amber stop codons. Data rearranged from Figure 4.

After prescreening the aaRS enzymes and ncAA substrates, a concentration dependent screening was performed with the most efficient ncAAs (O‑propargyl- and O-allyl-tyrosine) (**Figure S4**). This showed that reporter production was slightly more efficient with *Mj*ONBYRS than with *Mj*PCNFRS. Since background suppression (reporter signals generated without ncAA addition) is significantly lower with *Mj*ONBYRS, the latter was selected to be the preferred aaRS enzyme.


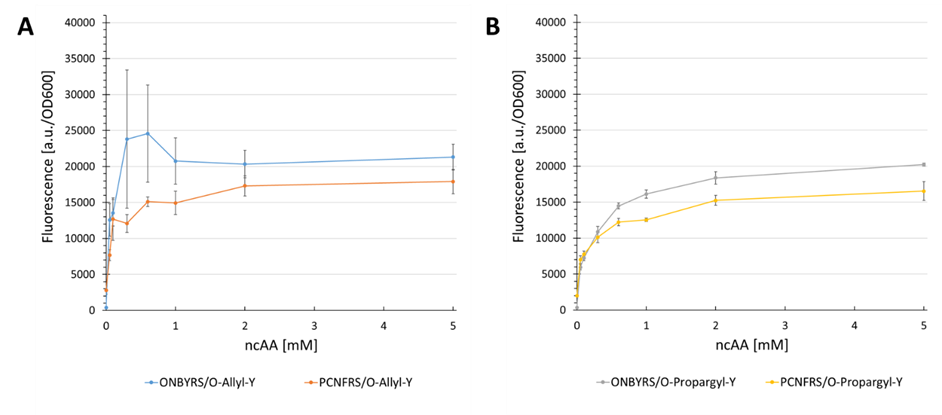


Figure S4. Concentration dependent protein production for *Mj*PCNFRS and *Mj*ONBYRS with the ncAAs A) O‑allyl-tyrosine and B) O-propargyl-tyrosine. Measured fluorescence intensity of intact *E. coli* BL21(DE3) cells expressing the SUMO-sfGFP(R2 amber) reporter. Endpoint measurements after 24 h with different ncAA concentrations (0.025, 0.05, 0.1, 0.3, 1, 2, and 5 mM). Data (incl. standard deviation) represent the mean of three biological replicates.


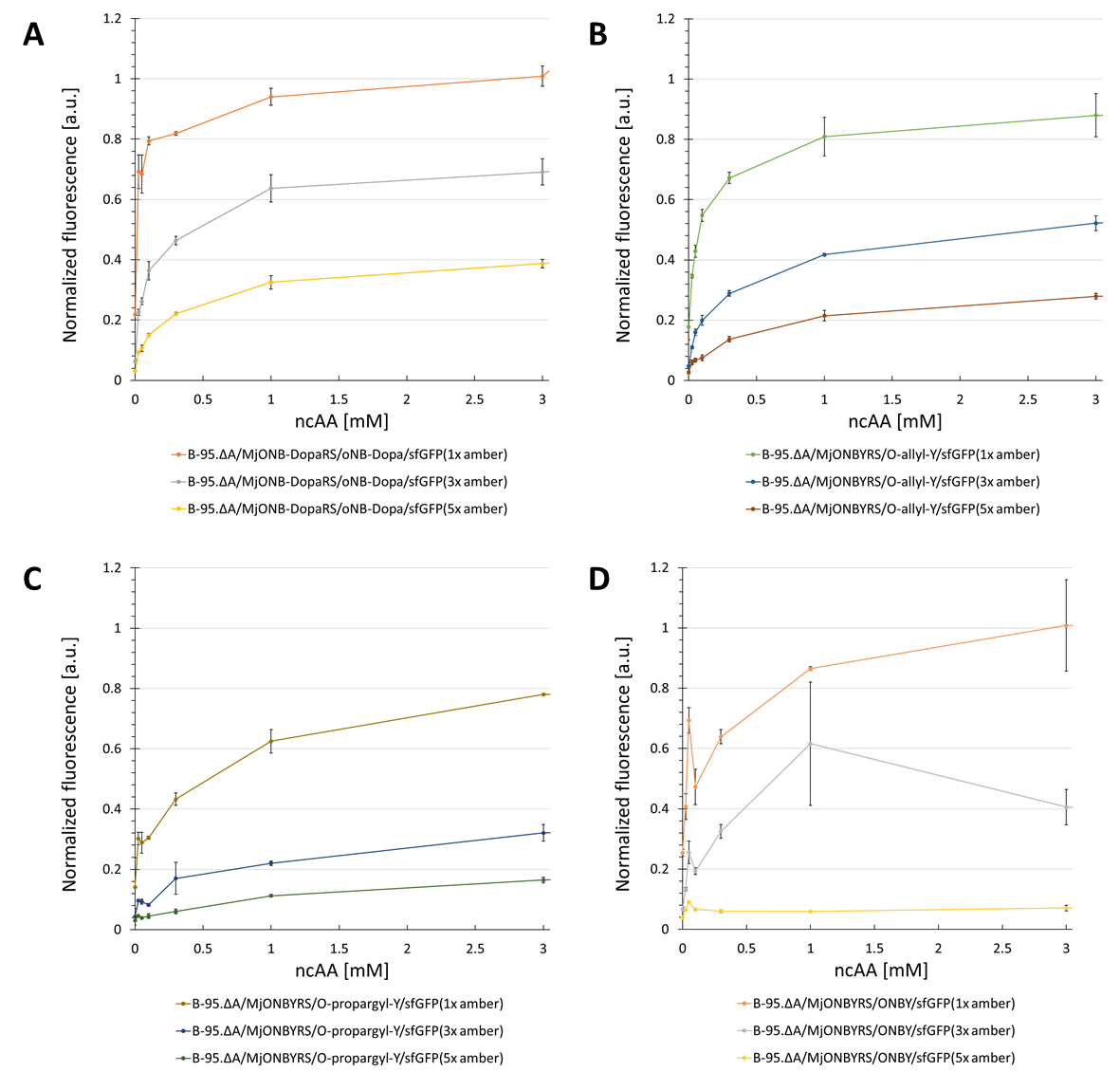


Figure S5: Concentration-dependent unnatural protein production with A) *Mj*ONB-DopaRS, B,C and D) *Mj*ONBYRS. The protein production host for reporter and aaRS co-expression was B-95.ΔA. Endpoint measurements for ncAA concentrations of 0.025, 0.05, 0.1, 0.3, 1 and 3 mM. Fluorescence values were normalized to the values for the wild-type sfGFP reporter constructs (without an in-frame stop codon). A value of 1 thus indicates a protein production level equivalent to wild-type.


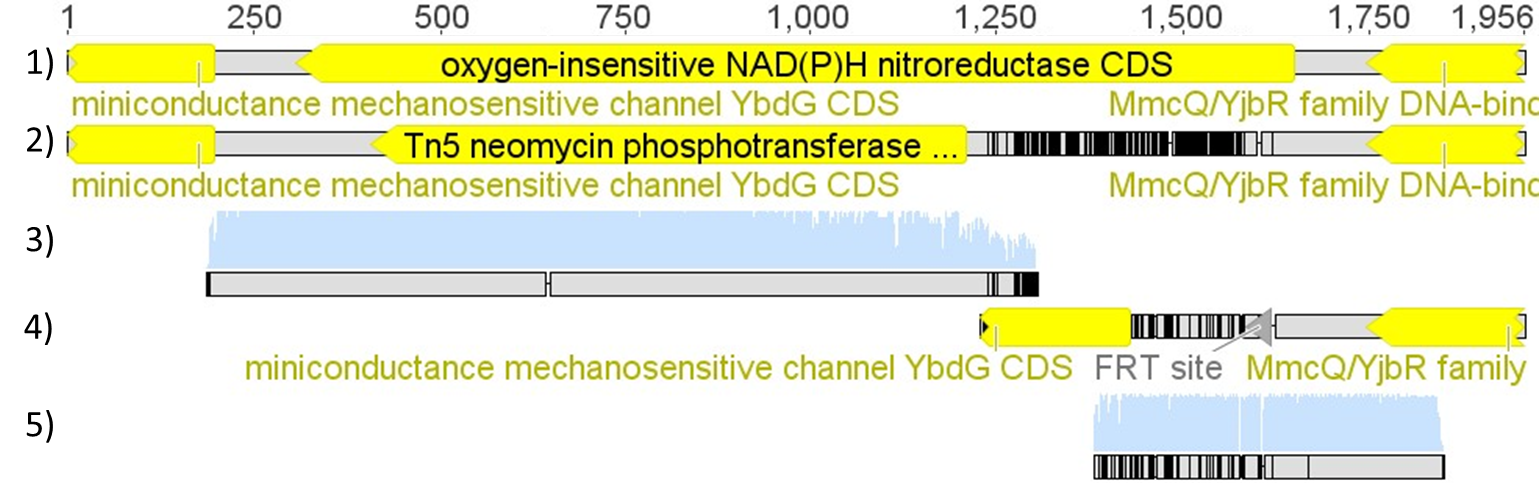


Figure S6. Confirmation of the nitroreductase gene knockout. Sequence analysis of bacterial strains harboring the neomycin resistance cassette and successor strains lacking the cassette, with the wild-type genomic *nfsB* target locus 1) as reference. 2) The corresponding locus wherein *nfsB* is replaced by the neomycin resistance cassette. 3) The corresponding PCR product Sanger sequencing result, experimentally obtained from the kanamycin resistant intermediate knockout strain. 4) Expected locus after genomic removal (“flip out”) of the neomycin resistance cassette, which leaves a FRT site (“scar”). 5) The corresponding sequencing result obtained from the kanamycin sensitive knockout strain. Mismatching sequence regions (where changes occurred as compared to the theoretical/reference sequence) are indicated in black, matches (where e.g. flanking regions remain unaltered) are shown in grey.


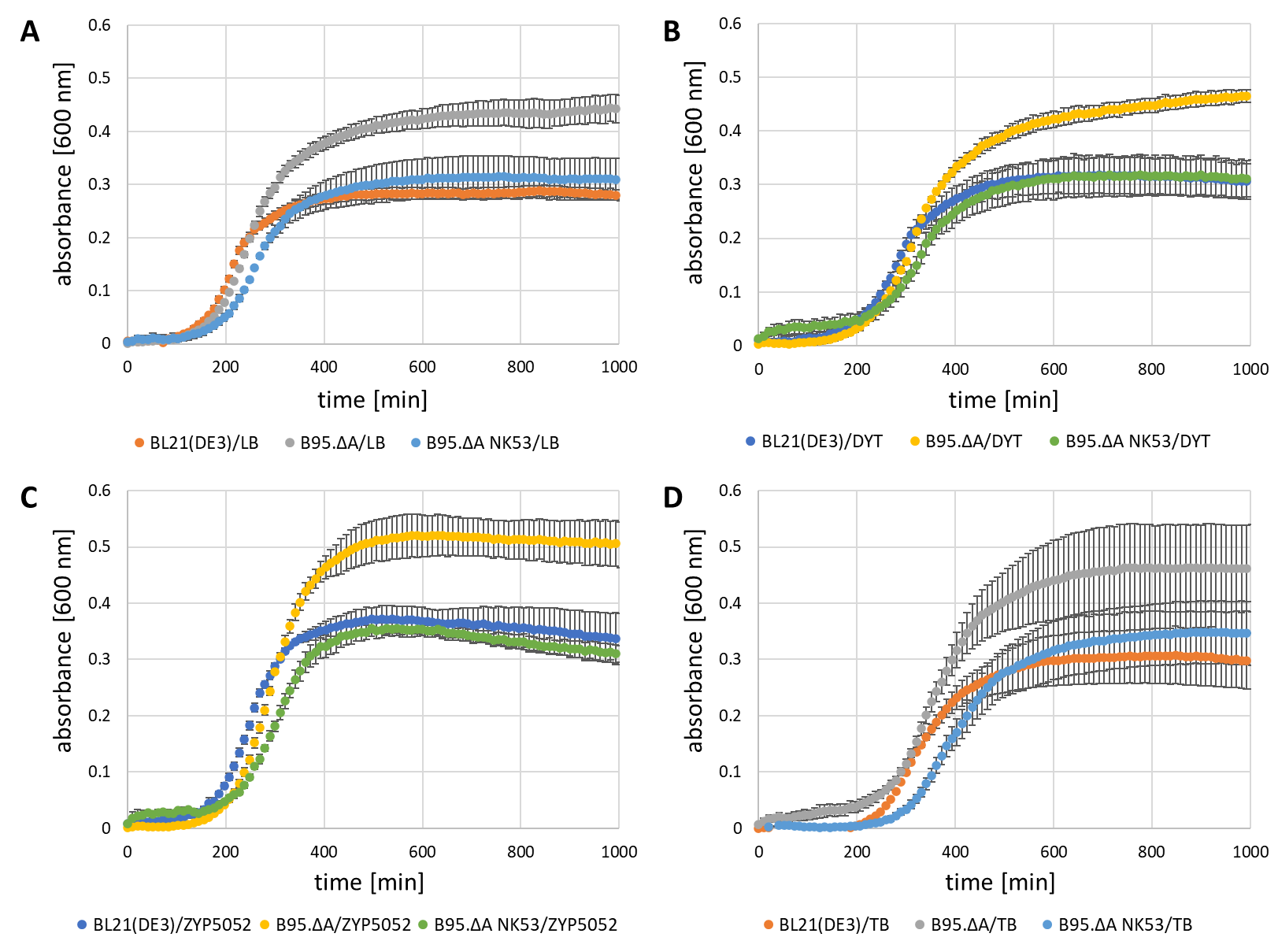


Figure S7. Growth curves of bacterial strains BL21(DE3), B-95.ΔA and B-95.ΔA NK53 with different growth media. A) LB medium, B) DYT medium, C) ZYP-5052 medium and D) TB medium. Cell growth was determined in indicated media in a 24-well plate format. OD_600_ measurements were performed using biological triplicates. Error bars represent the standard deviation of these triplicates.


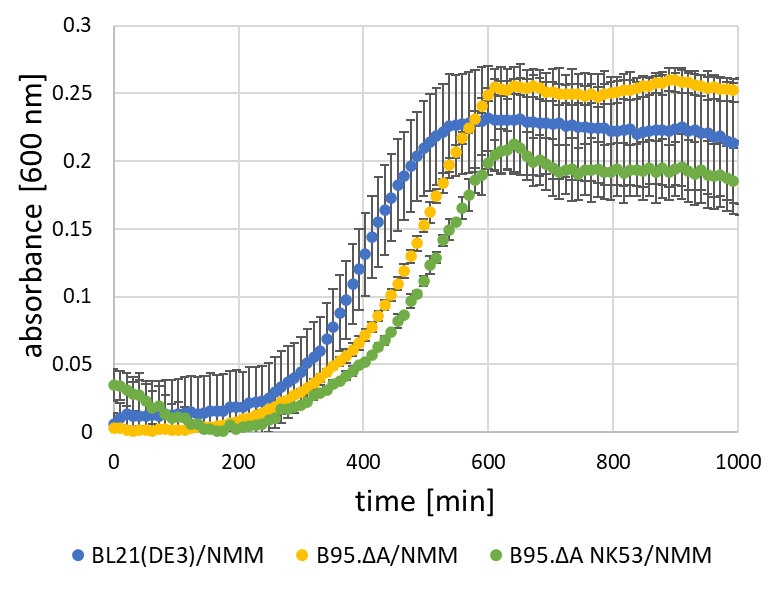


Figure S8. Growth curves of BL21(DE3), B-95.ΔA and B-95.ΔA NK53. Bacterial growth was determined in chemically defined new minimal media (NMM) (Budisa et al., 1995) in a 24-well plate format. OD_600_ measurements were performed using biological triplicates. Error bars are the standard deviation of these triplicates.


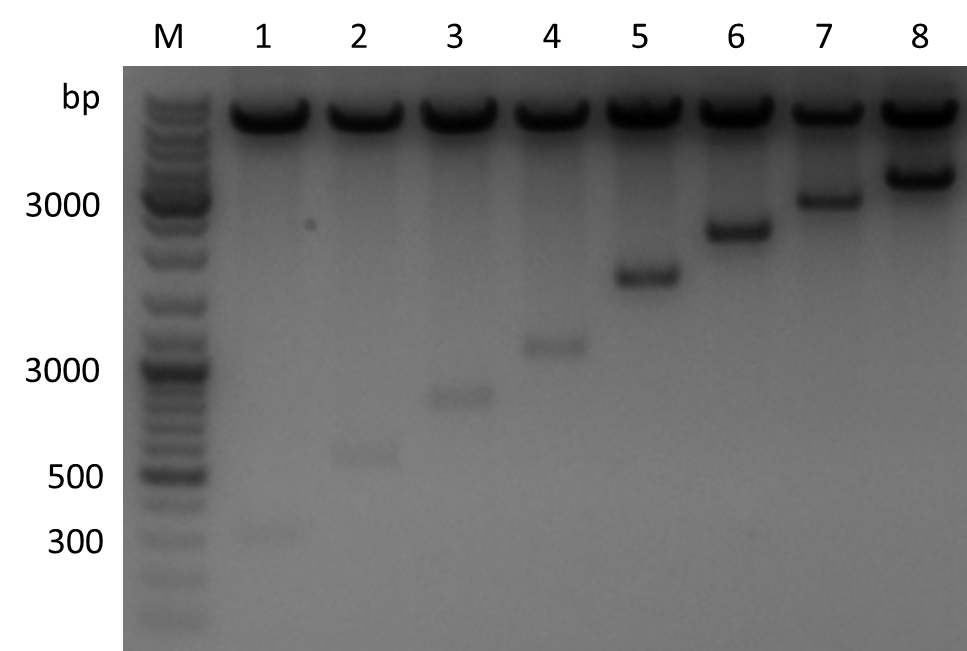


Figure S9: Agarose gel electrophoresis of an analytical plasmid DNA restriction digest (“test digest”) of pET-28a_ELP-sfGFP containing different numbers of ELP repeat constructs with in-frame amber stop codons (5-60). Restriction enzymes were BglI and XbaI. Lanes: M = size marker, lane 1 = 5, 2 = 10, 3 = 15, 4 = 20, 5 = 30, 6 = 40, 7 = 50, 8 = 60 stop codons, respectively. The size of the plasmid vector backbone fragments (top bands in lanes 1-8) is 5943 bp. 1% agarose gel in TAE-buffer.

## ESI-MS Spectra


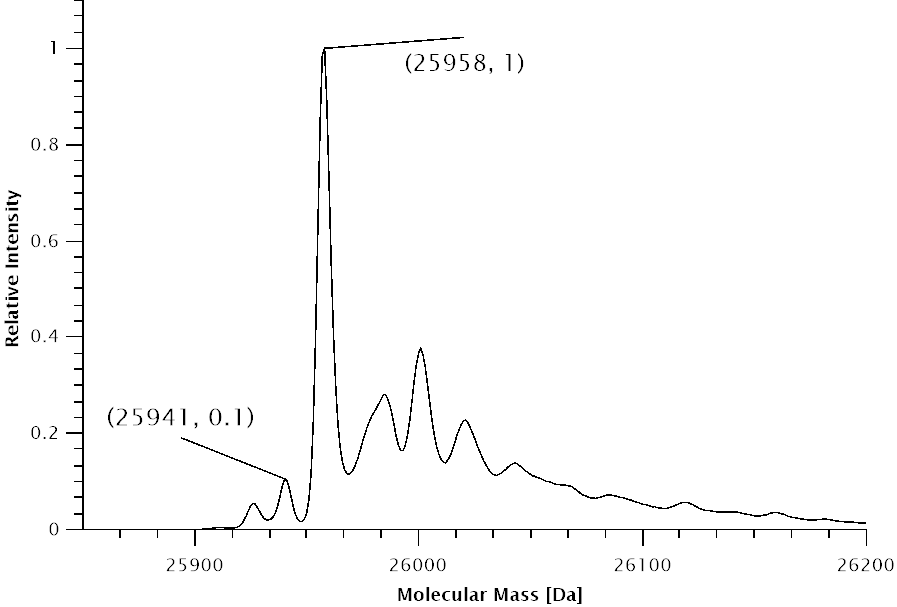


Figure S10. Deconvoluted ESI-MS spectrum of amilCP(Y66(ONBY)-His_6_. Protein production was performed in *E. coli* B-95.ΔA with co-expression of *Mj*ONB-DopaRS. Expected protein mass with non-maturated amilCP chromophore and unreduced ncAA nitro group: 25957.9 Da. Observed mass: 25958 Da. The 25941 Da by-product peak (relative intensity = 0.1) could represent maturated amilCP arising from background suppression with Tyr.


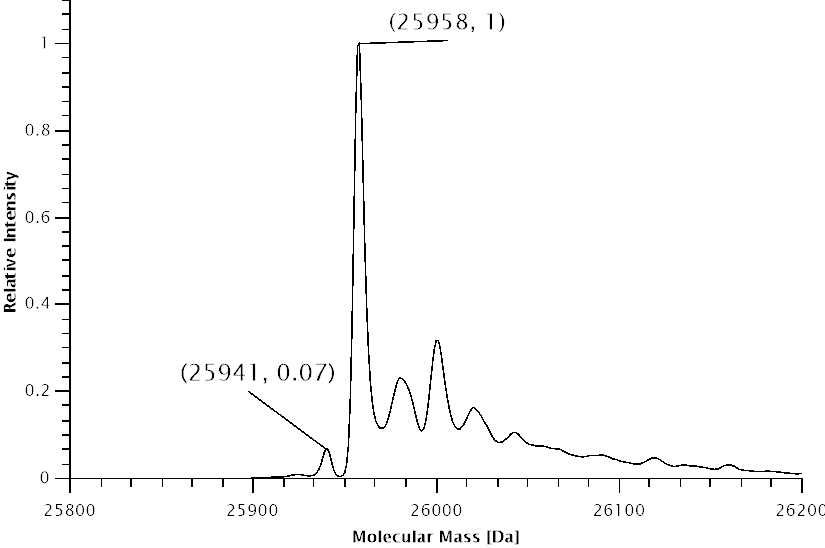


Figure S11. Deconvoluted ESI-MS spectrum of amilCP(Y66(ONBY)-His_6_. Protein production was performed in *E. coli* B-95.ΔA NK53 with co-expression of *Mj*ONB-DopaRS. Expected protein mass with non-maturated amilCP chromophore and unreduced ncAA nitro group: 25957.9 Da. Observed mass: 25958 Da. The 25941 Da by-product peak (relative intensity = 0.07) could represent maturated amilCP arising from background suppression with Tyr .

## DNA Sequences and Details of ELP-sfGFP Constructs

Details on the ELP-sfGFP fusion protein constructs used to generate the data shown in Figure 4. ELP constructs termed “normal” are based on ELP(5x amber), while the others termed “alternating” are based on ELP(8x amber).

**Table S1: ELP-sfGFP fusion protein construct details.**

|  |  |  |  |  |
| --- | --- | --- | --- | --- |
|  | **construct pair** | **N-terminal ELP length [aa]  until fluorescence reporter (RKGEE…)** | **hydrophobic residues in ELP part**  # | % |
| normal | 5x amber | 95 | 88 | 92.6% |
| alternating | 8x amber |  | 85 | 89.5% |
|  |  |  |  |  |
| normal | 10x amber | 175 | 163 | 93.1% |
| alternating | 16x amber |  | 157 | 89.7% |
|  |  |  |  |  |
| normal | 15x amber | 255 | 238 | 93.3% |
| alternating | 24x amber |  | 229 | 89.8% |
|  |  |  |  |  |
| normal | 20x amber | 335 | 313 | 93.4% |
| alternating | 32x amber |  | 301 | 89.9% |
|  |  |  |  |  |
| normal | 30x amber | 495 | 463 | 93.5% |
| alternating | 48x amber |  | 445 | 89.9% |
|  |  |  |  |  |
| normal | 40x amber | 655 | 613 | 93.6% |
| alternating | 64x amber |  | 589 | 89.9% |
|  |  |  |  |  |
|  |  |  | averages |  |
|  |  |  | normal | 93.3% |
|  |  |  | alternating | 89.8% |

In the following sequences, MSK stands for the N-terminal tripeptide encoded by the expression vector. The ELP monomer repeat sequence as part of the “normal” constructs is the following:


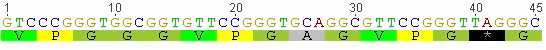


Three repeats of the (VPGX_n_G) sequence are shown here, with X_3_ as site of ribosomal non-canonical amino acid incorporation by suppression of the amber stop codon and further X_1_=G, X_2_=A. The full-length DNA sequences of the fusion protein genes are as follows:

Construct used for ESI-MS measurements, created initially without RDL:

MSK-ELP(5x amber)-sfGFP-His

ATGAGCAAAGGTGTCCCGGGTGGCGGTGTTCCGGGTGCAGGCGTTCCGGGTTAGGGCGTGCCGGGCGGCGGTGTTCCGGGTGCTGGTGTGCCGGGCTAGGGTGTCCCGGGTGGCGGTGTGCCGGGCGCAGGTGTCCCGGGTTAGGGTGTTCCGGGCGGCGGTGTCCCGGGTGCAGGTGTGCCGGGCTAGGGTGTGCCGGGCGGCGGTGTCCCGGGTGCAGGTGTGCCGGGCTAGGGTCCGGGTGGCGGTGGCCGTAAAGGCGAAGAGCTGTTCACTGGTGTCGTCCCTATTCTGGTGGAACTGGATGGTGATGTCAACGGTCATAAGTTTTCCGTGCGTGGCGAGGGTGAAGGTGACGCAACTAATGGTAAACTGACGCTGAAGTTCATCTGTACTACTGGTAAACTGCCGGTACCTTGGCCGACTCTGGTAACGACGCTGACTTATGGTGTTCAGTGCTTTGCTCGTTATCCGGACCATATGAAGCAGCATGACTTCTTCAAGTCCGCCATGCCGGAAGGCTATGTGCAGGAACGCACGATTTCCTTTAAGGATGACGGCACGTACAAAACGCGTGCGGAAGTGAAATTTGAAGGCGATACCCTGGTAAACCGCATTGAGCTGAAAGGCATTGACTTTAAAGAAGACGGCAATATCCTGGGCCATAAGCTGGAATACAATTTTAACAGCCACAATGTTTACATCACCGCCGATAAACAAAAAAATGGCATTAAAGCGAATTTTAAAATTCGCCACAACGTGGAGGATGGCAGCGTGCAGCTGGCTGATCACTACCAGCAAAACACTCCAATCGGTGATGGTCCTGTTCTGCTGCCAGACAATCACTATCTGAGCACGCAAAGCGTTCTGTCTAAAGATCCGAACGAGAAACGCGATCATATGGTTCTGCTGGAGTTCGTAACCGCAGCGGGCATCACGCATGGTATGGATGAACTGTACAAAGGCAGCCATCATCATCATCATCACTAA

Reporter constructs used for fluorescence assays:

MSK-ELP(5x amber)-sfGFP-His

ATGAGCAAAGGTGGGCCGGGCGTGGGTGTCCCGGGTGGCGGTGTTCCGGGTGCAGGCGTTCCGGGTTAGGGCGTGCCGGGCGGCGGTGTTCCGGGTGCTGGTGTGCCGGGCTAGGGTGTCCCGGGTGGCGGTGTGCCGGGCGCAGGTGTCCCGGGTTAGGGTGTTCCGGGCGGCGGTGTCCCGGGTGCAGGTGTGCCGGGCTAGGGTGTGCCGGGCGGCGGTGTCCCGGGTGCAGGTGTGCCGGGCTAGGGTGTGCCGGGCTGGCTGGGCCCGGGCGGTGGCGGTCGTAAAGGCGAAGAGCTGTTCACTGGTGTCGTCCCTATTCTGGTGGAACTGGATGGTGATGTCAACGGTCATAAGTTTTCCGTGCGTGGCGAGGGTGAAGGTGACGCAACTAATGGTAAACTGACGCTGAAGTTCATCTGTACTACTGGTAAACTGCCGGTACCTTGGCCGACTCTGGTAACGACGCTGACTTATGGTGTTCAGTGCTTTGCTCGTTATCCGGACCATATGAAGCAGCATGACTTCTTCAAGTCCGCCATGCCGGAAGGCTATGTGCAGGAACGCACGATTTCCTTTAAGGATGACGGCACGTACAAAACGCGTGCGGAAGTGAAATTTGAAGGCGATACCCTGGTAAACCGCATTGAGCTGAAAGGCATTGACTTTAAAGAAGACGGCAATATCCTGGGCCATAAGCTGGAATACAATTTTAACAGCCACAATGTTTACATCACCGCCGATAAACAAAAAAATGGCATTAAAGCGAATTTTAAAATTCGCCACAACGTGGAGGATGGCAGCGTGCAGCTGGCTGATCACTACCAGCAAAACACTCCAATCGGTGATGGTCCTGTTCTGCTGCCAGACAATCACTATCTGAGCACGCAAAGCGTTCTGTCTAAAGATCCGAACGAGAAACGCGATCATATGGTTCTGCTGGAGTTCGTAACCGCAGCGGGCATCACGCATGGTATGGATGAACTGTACAAAGGCAGCCATCATCATCATCATCACTAA

MSK-ELP(10x amber)-sfGFP-His

ATGAGCAAAGGTGGGCCGGGCGTGGGTGTCCCGGGTGGCGGTGTTCCGGGTGCAGGCGTTCCGGGTTAGGGCGTGCCGGGCGGCGGTGTTCCGGGTGCTGGTGTGCCGGGCTAGGGTGTCCCGGGTGGCGGTGTGCCGGGCGCAGGTGTCCCGGGTTAGGGTGTTCCGGGCGGCGGTGTCCCGGGTGCAGGTGTGCCGGGCTAGGGTGTGCCGGGCGGCGGTGTCCCGGGTGCAGGTGTGCCGGGCTAGGGTGTGCCGGGCGTGGGTGTCCCGGGTGGCGGTGTTCCGGGTGCAGGCGTTCCGGGTTAGGGCGTGCCGGGCGGCGGTGTTCCGGGTGCTGGTGTGCCGGGCTAGGGTGTCCCGGGTGGCGGTGTGCCGGGCGCAGGTGTCCCGGGTTAGGGTGTTCCGGGCGGCGGTGTCCCGGGTGCAGGTGTGCCGGGCTAGGGTGTGCCGGGCGGCGGTGTCCCGGGTGCAGGTGTGCCGGGCTAGGGTGTGCCGGGCTGGCTGGGCCCGGGCGGTGGCGGTCGTAAAGGCGAAGAGCTGTTCACTGGTGTCGTCCCTATTCTGGTGGAACTGGATGGTGATGTCAACGGTCATAAGTTTTCCGTGCGTGGCGAGGGTGAAGGTGACGCAACTAATGGTAAACTGACGCTGAAGTTCATCTGTACTACTGGTAAACTGCCGGTACCTTGGCCGACTCTGGTAACGACGCTGACTTATGGTGTTCAGTGCTTTGCTCGTTATCCGGACCATATGAAGCAGCATGACTTCTTCAAGTCCGCCATGCCGGAAGGCTATGTGCAGGAACGCACGATTTCCTTTAAGGATGACGGCACGTACAAAACGCGTGCGGAAGTGAAATTTGAAGGCGATACCCTGGTAAACCGCATTGAGCTGAAAGGCATTGACTTTAAAGAAGACGGCAATATCCTGGGCCATAAGCTGGAATACAATTTTAACAGCCACAATGTTTACATCACCGCCGATAAACAAAAAAATGGCATTAAAGCGAATTTTAAAATTCGCCACAACGTGGAGGATGGCAGCGTGCAGCTGGCTGATCACTACCAGCAAAACACTCCAATCGGTGATGGTCCTGTTCTGCTGCCAGACAATCACTATCTGAGCACGCAAAGCGTTCTGTCTAAAGATCCGAACGAGAAACGCGATCATATGGTTCTGCTGGAGTTCGTAACCGCAGCGGGCATCACGCATGGTATGGATGAACTGTACAAAGGCAGCCATCATCATCATCATCACTAA

MSK-ELP(15x amber)-sfGFP-His

ATGAGCAAAGGTGGGCCGGGCGTGGGTGTCCCGGGTGGCGGTGTTCCGGGTGCAGGCGTTCCGGGTTAGGGCGTGCCGGGCGGCGGTGTTCCGGGTGCTGGTGTGCCGGGCTAGGGTGTCCCGGGTGGCGGTGTGCCGGGCGCAGGTGTCCCGGGTTAGGGTGTTCCGGGCGGCGGTGTCCCGGGTGCAGGTGTGCCGGGCTAGGGTGTGCCGGGCGGCGGTGTCCCGGGTGCAGGTGTGCCGGGCTAGGGTGTGCCGGGCGTGGGTGTCCCGGGTGGCGGTGTTCCGGGTGCAGGCGTTCCGGGTTAGGGCGTGCCGGGCGGCGGTGTTCCGGGTGCTGGTGTGCCGGGCTAGGGTGTCCCGGGTGGCGGTGTGCCGGGCGCAGGTGTCCCGGGTTAGGGTGTTCCGGGCGGCGGTGTCCCGGGTGCAGGTGTGCCGGGCTAGGGTGTGCCGGGCGGCGGTGTCCCGGGTGCAGGTGTGCCGGGCTAGGGTGTGCCGGGCGTGGGTGTCCCGGGTGGCGGTGTTCCGGGTGCAGGCGTTCCGGGTTAGGGCGTGCCGGGCGGCGGTGTTCCGGGTGCTGGTGTGCCGGGCTAGGGTGTCCCGGGTGGCGGTGTGCCGGGCGCAGGTGTCCCGGGTTAGGGTGTTCCGGGCGGCGGTGTCCCGGGTGCAGGTGTGCCGGGCTAGGGTGTGCCGGGCGGCGGTGTCCCGGGTGCAGGTGTGCCGGGCTAGGGTGTGCCGGGCTGGCTGGGCCCGGGCGGTGGCGGTCGTAAAGGCGAAGAGCTGTTCACTGGTGTCGTCCCTATTCTGGTGGAACTGGATGGTGATGTCAACGGTCATAAGTTTTCCGTGCGTGGCGAGGGTGAAGGTGACGCAACTAATGGTAAACTGACGCTGAAGTTCATCTGTACTACTGGTAAACTGCCGGTACCTTGGCCGACTCTGGTAACGACGCTGACTTATGGTGTTCAGTGCTTTGCTCGTTATCCGGACCATATGAAGCAGCATGACTTCTTCAAGTCCGCCATGCCGGAAGGCTATGTGCAGGAACGCACGATTTCCTTTAAGGATGACGGCACGTACAAAACGCGTGCGGAAGTGAAATTTGAAGGCGATACCCTGGTAAACCGCATTGAGCTGAAAGGCATTGACTTTAAAGAAGACGGCAATATCCTGGGCCATAAGCTGGAATACAATTTTAACAGCCACAATGTTTACATCACCGCCGATAAACAAAAAAATGGCATTAAAGCGAATTTTAAAATTCGCCACAACGTGGAGGATGGCAGCGTGCAGCTGGCTGATCACTACCAGCAAAACACTCCAATCGGTGATGGTCCTGTTCTGCTGCCAGACAATCACTATCTGAGCACGCAAAGCGTTCTGTCTAAAGATCCGAACGAGAAACGCGATCATATGGTTCTGCTGGAGTTCGTAACCGCAGCGGGCATCACGCATGGTATGGATGAACTGTACAAAGGCAGCCATCATCATCATCATCACTAA

MSK-ELP(20x amber)-sfGFP-His

ATGAGCAAAGGTGGGCCGGGCGTGGGTGTCCCGGGTGGCGGTGTTCCGGGTGCAGGCGTTCCGGGTTAGGGCGTGCCGGGCGGCGGTGTTCCGGGTGCTGGTGTGCCGGGCTAGGGTGTCCCGGGTGGCGGTGTGCCGGGCGCAGGTGTCCCGGGTTAGGGTGTTCCGGGCGGCGGTGTCCCGGGTGCAGGTGTGCCGGGCTAGGGTGTGCCGGGCGGCGGTGTCCCGGGTGCAGGTGTGCCGGGCTAGGGTGTGCCGGGCGTGGGTGTCCCGGGTGGCGGTGTTCCGGGTGCAGGCGTTCCGGGTTAGGGCGTGCCGGGCGGCGGTGTTCCGGGTGCTGGTGTGCCGGGCTAGGGTGTCCCGGGTGGCGGTGTGCCGGGCGCAGGTGTCCCGGGTTAGGGTGTTCCGGGCGGCGGTGTCCCGGGTGCAGGTGTGCCGGGCTAGGGTGTGCCGGGCGGCGGTGTCCCGGGTGCAGGTGTGCCGGGCTAGGGTGTGCCGGGCGTGGGTGTCCCGGGTGGCGGTGTTCCGGGTGCAGGCGTTCCGGGTTAGGGCGTGCCGGGCGGCGGTGTTCCGGGTGCTGGTGTGCCGGGCTAGGGTGTCCCGGGTGGCGGTGTGCCGGGCGCAGGTGTCCCGGGTTAGGGTGTTCCGGGCGGCGGTGTCCCGGGTGCAGGTGTGCCGGGCTAGGGTGTGCCGGGCGGCGGTGTCCCGGGTGCAGGTGTGCCGGGCTAGGGTGTGCCGGGCGTGGGTGTCCCGGGTGGCGGTGTTCCGGGTGCAGGCGTTCCGGGTTAGGGCGTGCCGGGCGGCGGTGTTCCGGGTGCTGGTGTGCCGGGCTAGGGTGTCCCGGGTGGCGGTGTGCCGGGCGCAGGTGTCCCGGGTTAGGGTGTTCCGGGCGGCGGTGTCCCGGGTGCAGGTGTGCCGGGCTAGGGTGTGCCGGGCGGCGGTGTCCCGGGTGCAGGTGTGCCGGGCTAGGGTGTGCCGGGCTGGCTGGGCCCGGGCGGTGGCGGTCGTAAAGGCGAAGAGCTGTTCACTGGTGTCGTCCCTATTCTGGTGGAACTGGATGGTGATGTCAACGGTCATAAGTTTTCCGTGCGTGGCGAGGGTGAAGGTGACGCAACTAATGGTAAACTGACGCTGAAGTTCATCTGTACTACTGGTAAACTGCCGGTACCTTGGCCGACTCTGGTAACGACGCTGACTTATGGTGTTCAGTGCTTTGCTCGTTATCCGGACCATATGAAGCAGCATGACTTCTTCAAGTCCGCCATGCCGGAAGGCTATGTGCAGGAACGCACGATTTCCTTTAAGGATGACGGCACGTACAAAACGCGTGCGGAAGTGAAATTTGAAGGCGATACCCTGGTAAACCGCATTGAGCTGAAAGGCATTGACTTTAAAGAAGACGGCAATATCCTGGGCCATAAGCTGGAATACAATTTTAACAGCCACAATGTTTACATCACCGCCGATAAACAAAAAAATGGCATTAAAGCGAATTTTAAAATTCGCCACAACGTGGAGGATGGCAGCGTGCAGCTGGCTGATCACTACCAGCAAAACACTCCAATCGGTGATGGTCCTGTTCTGCTGCCAGACAATCACTATCTGAGCACGCAAAGCGTTCTGTCTAAAGATCCGAACGAGAAACGCGATCATATGGTTCTGCTGGAGTTCGTAACCGCAGCGGGCATCACGCATGGTATGGATGAACTGTACAAAGGCAGCCATCATCATCATCATCACTAA

MSK-ELP(30x amber)-sfGFP-His

ATGAGCAAAGGTGGGCCGGGCGTGGGTGTCCCGGGTGGCGGTGTTCCGGGTGCAGGCGTTCCGGGTTAGGGCGTGCCGGGCGGCGGTGTTCCGGGTGCTGGTGTGCCGGGCTAGGGTGTCCCGGGTGGCGGTGTGCCGGGCGCAGGTGTCCCGGGTTAGGGTGTTCCGGGCGGCGGTGTCCCGGGTGCAGGTGTGCCGGGCTAGGGTGTGCCGGGCGGCGGTGTCCCGGGTGCAGGTGTGCCGGGCTAGGGTGTGCCGGGCGTGGGTGTCCCGGGTGGCGGTGTTCCGGGTGCAGGCGTTCCGGGTTAGGGCGTGCCGGGCGGCGGTGTTCCGGGTGCTGGTGTGCCGGGCTAGGGTGTCCCGGGTGGCGGTGTGCCGGGCGCAGGTGTCCCGGGTTAGGGTGTTCCGGGCGGCGGTGTCCCGGGTGCAGGTGTGCCGGGCTAGGGTGTGCCGGGCGGCGGTGTCCCGGGTGCAGGTGTGCCGGGCTAGGGTGTGCCGGGCGTGGGTGTCCCGGGTGGCGGTGTTCCGGGTGCAGGCGTTCCGGGTTAGGGCGTGCCGGGCGGCGGTGTTCCGGGTGCTGGTGTGCCGGGCTAGGGTGTCCCGGGTGGCGGTGTGCCGGGCGCAGGTGTCCCGGGTTAGGGTGTTCCGGGCGGCGGTGTCCCGGGTGCAGGTGTGCCGGGCTAGGGTGTGCCGGGCGGCGGTGTCCCGGGTGCAGGTGTGCCGGGCTAGGGTGTGCCGGGCGTGGGTGTCCCGGGTGGCGGTGTTCCGGGTGCAGGCGTTCCGGGTTAGGGCGTGCCGGGCGGCGGTGTTCCGGGTGCTGGTGTGCCGGGCTAGGGTGTCCCGGGTGGCGGTGTGCCGGGCGCAGGTGTCCCGGGTTAGGGTGTTCCGGGCGGCGGTGTCCCGGGTGCAGGTGTGCCGGGCTAGGGTGTGCCGGGCGGCGGTGTCCCGGGTGCAGGTGTGCCGGGCTAGGGTGTGCCGGGCGTGGGTGTCCCGGGTGGCGGTGTTCCGGGTGCAGGCGTTCCGGGTTAGGGCGTGCCGGGCGGCGGTGTTCCGGGTGCTGGTGTGCCGGGCTAGGGTGTCCCGGGTGGCGGTGTGCCGGGCGCAGGTGTCCCGGGTTAGGGTGTTCCGGGCGGCGGTGTCCCGGGTGCAGGTGTGCCGGGCTAGGGTGTGCCGGGCGGCGGTGTCCCGGGTGCAGGTGTGCCGGGCTAGGGTGTGCCGGGCGTGGGTGTCCCGGGTGGCGGTGTTCCGGGTGCAGGCGTTCCGGGTTAGGGCGTGCCGGGCGGCGGTGTTCCGGGTGCTGGTGTGCCGGGCTAGGGTGTCCCGGGTGGCGGTGTGCCGGGCGCAGGTGTCCCGGGTTAGGGTGTTCCGGGCGGCGGTGTCCCGGGTGCAGGTGTGCCGGGCTAGGGTGTGCCGGGCGGCGGTGTCCCGGGTGCAGGTGTGCCGGGCTAGGGTGTGCCGGGCTGGCTGGGCCCGGGCGGTGGCGGTCGTAAAGGCGAAGAGCTGTTCACTGGTGTCGTCCCTATTCTGGTGGAACTGGATGGTGATGTCAACGGTCATAAGTTTTCCGTGCGTGGCGAGGGTGAAGGTGACGCAACTAATGGTAAACTGACGCTGAAGTTCATCTGTACTACTGGTAAACTGCCGGTACCTTGGCCGACTCTGGTAACGACGCTGACTTATGGTGTTCAGTGCTTTGCTCGTTATCCGGACCATATGAAGCAGCATGACTTCTTCAAGTCCGCCATGCCGGAAGGCTATGTGCAGGAACGCACGATTTCCTTTAAGGATGACGGCACGTACAAAACGCGTGCGGAAGTGAAATTTGAAGGCGATACCCTGGTAAACCGCATTGAGCTGAAAGGCATTGACTTTAAAGAAGACGGCAATATCCTGGGCCATAAGCTGGAATACAATTTTAACAGCCACAATGTTTACATCACCGCCGATAAACAAAAAAATGGCATTAAAGCGAATTTTAAAATTCGCCACAACGTGGAGGATGGCAGCGTGCAGCTGGCTGATCACTACCAGCAAAACACTCCAATCGGTGATGGTCCTGTTCTGCTGCCAGACAATCACTATCTGAGCACGCAAAGCGTTCTGTCTAAAGATCCGAACGAGAAACGCGATCATATGGTTCTGCTGGAGTTCGTAACCGCAGCGGGCATCACGCATGGTATGGATGAACTGTACAAAGGCAGCCATCATCATCATCATCACTAA

MSK-ELP(40x amber)-sfGFP-His

ATGAGCAAAGGTGGGCCGGGCGTGGGTGTCCCGGGTGGCGGTGTTCCGGGTGCAGGCGTTCCGGGTTAGGGCGTGCCGGGCGGCGGTGTTCCGGGTGCTGGTGTGCCGGGCTAGGGTGTCCCGGGTGGCGGTGTGCCGGGCGCAGGTGTCCCGGGTTAGGGTGTTCCGGGCGGCGGTGTCCCGGGTGCAGGTGTGCCGGGCTAGGGTGTGCCGGGCGGCGGTGTCCCGGGTGCAGGTGTGCCGGGCTAGGGTGTGCCGGGCGTGGGTGTCCCGGGTGGCGGTGTTCCGGGTGCAGGCGTTCCGGGTTAGGGCGTGCCGGGCGGCGGTGTTCCGGGTGCTGGTGTGCCGGGCTAGGGTGTCCCGGGTGGCGGTGTGCCGGGCGCAGGTGTCCCGGGTTAGGGTGTTCCGGGCGGCGGTGTCCCGGGTGCAGGTGTGCCGGGCTAGGGTGTGCCGGGCGGCGGTGTCCCGGGTGCAGGTGTGCCGGGCTAGGGTGTGCCGGGCGTGGGTGTCCCGGGTGGCGGTGTTCCGGGTGCAGGCGTTCCGGGTTAGGGCGTGCCGGGCGGCGGTGTTCCGGGTGCTGGTGTGCCGGGCTAGGGTGTCCCGGGTGGCGGTGTGCCGGGCGCAGGTGTCCCGGGTTAGGGTGTTCCGGGCGGCGGTGTCCCGGGTGCAGGTGTGCCGGGCTAGGGTGTGCCGGGCGGCGGTGTCCCGGGTGCAGGTGTGCCGGGCTAGGGTGTGCCGGGCGTGGGTGTCCCGGGTGGCGGTGTTCCGGGTGCAGGCGTTCCGGGTTAGGGCGTGCCGGGCGGCGGTGTTCCGGGTGCTGGTGTGCCGGGCTAGGGTGTCCCGGGTGGCGGTGTGCCGGGCGCAGGTGTCCCGGGTTAGGGTGTTCCGGGCGGCGGTGTCCCGGGTGCAGGTGTGCCGGGCTAGGGTGTGCCGGGCGGCGGTGTCCCGGGTGCAGGTGTGCCGGGCTAGGGTGTGCCGGGCGTGGGTGTCCCGGGTGGCGGTGTTCCGGGTGCAGGCGTTCCGGGTTAGGGCGTGCCGGGCGGCGGTGTTCCGGGTGCTGGTGTGCCGGGCTAGGGTGTCCCGGGTGGCGGTGTGCCGGGCGCAGGTGTCCCGGGTTAGGGTGTTCCGGGCGGCGGTGTCCCGGGTGCAGGTGTGCCGGGCTAGGGTGTGCCGGGCGGCGGTGTCCCGGGTGCAGGTGTGCCGGGCTAGGGTGTGCCGGGCGTGGGTGTCCCGGGTGGCGGTGTTCCGGGTGCAGGCGTTCCGGGTTAGGGCGTGCCGGGCGGCGGTGTTCCGGGTGCTGGTGTGCCGGGCTAGGGTGTCCCGGGTGGCGGTGTGCCGGGCGCAGGTGTCCCGGGTTAGGGTGTTCCGGGCGGCGGTGTCCCGGGTGCAGGTGTGCCGGGCTAGGGTGTGCCGGGCGGCGGTGTCCCGGGTGCAGGTGTGCCGGGCTAGGGTGTGCCGGGCGTGGGTGTCCCGGGTGGCGGTGTTCCGGGTGCAGGCGTTCCGGGTTAGGGCGTGCCGGGCGGCGGTGTTCCGGGTGCTGGTGTGCCGGGCTAGGGTGTCCCGGGTGGCGGTGTGCCGGGCGCAGGTGTCCCGGGTTAGGGTGTTCCGGGCGGCGGTGTCCCGGGTGCAGGTGTGCCGGGCTAGGGTGTGCCGGGCGGCGGTGTCCCGGGTGCAGGTGTGCCGGGCTAGGGTGTGCCGGGCGTGGGTGTCCCGGGTGGCGGTGTTCCGGGTGCAGGCGTTCCGGGTTAGGGCGTGCCGGGCGGCGGTGTTCCGGGTGCTGGTGTGCCGGGCTAGGGTGTCCCGGGTGGCGGTGTGCCGGGCGCAGGTGTCCCGGGTTAGGGTGTTCCGGGCGGCGGTGTCCCGGGTGCAGGTGTGCCGGGCTAGGGTGTGCCGGGCGGCGGTGTCCCGGGTGCAGGTGTGCCGGGCTAGGGTGTGCCGGGCTGGCTGGGCCCGGGCGGTGGCGGTCGTAAAGGCGAAGAGCTGTTCACTGGTGTCGTCCCTATTCTGGTGGAACTGGATGGTGATGTCAACGGTCATAAGTTTTCCGTGCGTGGCGAGGGTGAAGGTGACGCAACTAATGGTAAACTGACGCTGAAGTTCATCTGTACTACTGGTAAACTGCCGGTACCTTGGCCGACTCTGGTAACGACGCTGACTTATGGTGTTCAGTGCTTTGCTCGTTATCCGGACCATATGAAGCAGCATGACTTCTTCAAGTCCGCCATGCCGGAAGGCTATGTGCAGGAACGCACGATTTCCTTTAAGGATGACGGCACGTACAAAACGCGTGCGGAAGTGAAATTTGAAGGCGATACCCTGGTAAACCGCATTGAGCTGAAAGGCATTGACTTTAAAGAAGACGGCAATATCCTGGGCCATAAGCTGGAATACAATTTTAACAGCCACAATGTTTACATCACCGCCGATAAACAAAAAAATGGCATTAAAGCGAATTTTAAAATTCGCCACAACGTGGAGGATGGCAGCGTGCAGCTGGCTGATCACTACCAGCAAAACACTCCAATCGGTGATGGTCCTGTTCTGCTGCCAGACAATCACTATCTGAGCACGCAAAGCGTTCTGTCTAAAGATCCGAACGAGAAACGCGATCATATGGTTCTGCTGGAGTTCGTAACCGCAGCGGGCATCACGCATGGTATGGATGAACTGTACAAAGGCAGCCATCATCATCATCATCACTAA

MSK-ELP(50x amber)-sfGFP-His

ATGAGCAAAGGTGGGCCGGGCGTGGGTGTCCCGGGTGGCGGTGTTCCGGGTGCAGGCGTTCCGGGTTAGGGCGTGCCGGGCGGCGGTGTTCCGGGTGCTGGTGTGCCGGGCTAGGGTGTCCCGGGTGGCGGTGTGCCGGGCGCAGGTGTCCCGGGTTAGGGTGTTCCGGGCGGCGGTGTCCCGGGTGCAGGTGTGCCGGGCTAGGGTGTGCCGGGCGGCGGTGTCCCGGGTGCAGGTGTGCCGGGCTAGGGTGTGCCGGGCGTGGGTGTCCCGGGTGGCGGTGTTCCGGGTGCAGGCGTTCCGGGTTAGGGCGTGCCGGGCGGCGGTGTTCCGGGTGCTGGTGTGCCGGGCTAGGGTGTCCCGGGTGGCGGTGTGCCGGGCGCAGGTGTCCCGGGTTAGGGTGTTCCGGGCGGCGGTGTCCCGGGTGCAGGTGTGCCGGGCTAGGGTGTGCCGGGCGGCGGTGTCCCGGGTGCAGGTGTGCCGGGCTAGGGTGTGCCGGGCGTGGGTGTCCCGGGTGGCGGTGTTCCGGGTGCAGGCGTTCCGGGTTAGGGCGTGCCGGGCGGCGGTGTTCCGGGTGCTGGTGTGCCGGGCTAGGGTGTCCCGGGTGGCGGTGTGCCGGGCGCAGGTGTCCCGGGTTAGGGTGTTCCGGGCGGCGGTGTCCCGGGTGCAGGTGTGCCGGGCTAGGGTGTGCCGGGCGGCGGTGTCCCGGGTGCAGGTGTGCCGGGCTAGGGTGTGCCGGGCGTGGGTGTCCCGGGTGGCGGTGTTCCGGGTGCAGGCGTTCCGGGTTAGGGCGTGCCGGGCGGCGGTGTTCCGGGTGCTGGTGTGCCGGGCTAGGGTGTCCCGGGTGGCGGTGTGCCGGGCGCAGGTGTCCCGGGTTAGGGTGTTCCGGGCGGCGGTGTCCCGGGTGCAGGTGTGCCGGGCTAGGGTGTGCCGGGCGGCGGTGTCCCGGGTGCAGGTGTGCCGGGCTAGGGTGTGCCGGGCGTGGGTGTCCCGGGTGGCGGTGTTCCGGGTGCAGGCGTTCCGGGTTAGGGCGTGCCGGGCGGCGGTGTTCCGGGTGCTGGTGTGCCGGGCTAGGGTGTCCCGGGTGGCGGTGTGCCGGGCGCAGGTGTCCCGGGTTAGGGTGTTCCGGGCGGCGGTGTCCCGGGTGCAGGTGTGCCGGGCTAGGGTGTGCCGGGCGGCGGTGTCCCGGGTGCAGGTGTGCCGGGCTAGGGTGTGCCGGGCGTGGGTGTCCCGGGTGGCGGTGTTCCGGGTGCAGGCGTTCCGGGTTAGGGCGTGCCGGGCGGCGGTGTTCCGGGTGCTGGTGTGCCGGGCTAGGGTGTCCCGGGTGGCGGTGTGCCGGGCGCAGGTGTCCCGGGTTAGGGTGTTCCGGGCGGCGGTGTCCCGGGTGCAGGTGTGCCGGGCTAGGGTGTGCCGGGCGGCGGTGTCCCGGGTGCAGGTGTGCCGGGCTAGGGTGTGCCGGGCGTGGGTGTCCCGGGTGGCGGTGTTCCGGGTGCAGGCGTTCCGGGTTAGGGCGTGCCGGGCGGCGGTGTTCCGGGTGCTGGTGTGCCGGGCTAGGGTGTCCCGGGTGGCGGTGTGCCGGGCGCAGGTGTCCCGGGTTAGGGTGTTCCGGGCGGCGGTGTCCCGGGTGCAGGTGTGCCGGGCTAGGGTGTGCCGGGCGGCGGTGTCCCGGGTGCAGGTGTGCCGGGCTAGGGTGTGCCGGGCGTGGGTGTCCCGGGTGGCGGTGTTCCGGGTGCAGGCGTTCCGGGTTAGGGCGTGCCGGGCGGCGGTGTTCCGGGTGCTGGTGTGCCGGGCTAGGGTGTCCCGGGTGGCGGTGTGCCGGGCGCAGGTGTCCCGGGTTAGGGTGTTCCGGGCGGCGGTGTCCCGGGTGCAGGTGTGCCGGGCTAGGGTGTGCCGGGCGGCGGTGTCCCGGGTGCAGGTGTGCCGGGCTAGGGTGTGCCGGGCGTGGGTGTCCCGGGTGGCGGTGTTCCGGGTGCAGGCGTTCCGGGTTAGGGCGTGCCGGGCGGCGGTGTTCCGGGTGCTGGTGTGCCGGGCTAGGGTGTCCCGGGTGGCGGTGTGCCGGGCGCAGGTGTCCCGGGTTAGGGTGTTCCGGGCGGCGGTGTCCCGGGTGCAGGTGTGCCGGGCTAGGGTGTGCCGGGCGGCGGTGTCCCGGGTGCAGGTGTGCCGGGCTAGGGTGTGCCGGGCGTGGGTGTCCCGGGTGGCGGTGTTCCGGGTGCAGGCGTTCCGGGTTAGGGCGTGCCGGGCGGCGGTGTTCCGGGTGCTGGTGTGCCGGGCTAGGGTGTCCCGGGTGGCGGTGTGCCGGGCGCAGGTGTCCCGGGTTAGGGTGTTCCGGGCGGCGGTGTCCCGGGTGCAGGTGTGCCGGGCTAGGGTGTGCCGGGCGGCGGTGTCCCGGGTGCAGGTGTGCCGGGCTAGGGTGTGCCGGGCTGGCTGGGCCCGGGCGGTGGCGGTCGTAAAGGCGAAGAGCTGTTCACTGGTGTCGTCCCTATTCTGGTGGAACTGGATGGTGATGTCAACGGTCATAAGTTTTCCGTGCGTGGCGAGGGTGAAGGTGACGCAACTAATGGTAAACTGACGCTGAAGTTCATCTGTACTACTGGTAAACTGCCGGTACCTTGGCCGACTCTGGTAACGACGCTGACTTATGGTGTTCAGTGCTTTGCTCGTTATCCGGACCATATGAAGCAGCATGACTTCTTCAAGTCCGCCATGCCGGAAGGCTATGTGCAGGAACGCACGATTTCCTTTAAGGATGACGGCACGTACAAAACGCGTGCGGAAGTGAAATTTGAAGGCGATACCCTGGTAAACCGCATTGAGCTGAAAGGCATTGACTTTAAAGAAGACGGCAATATCCTGGGCCATAAGCTGGAATACAATTTTAACAGCCACAATGTTTACATCACCGCCGATAAACAAAAAAATGGCATTAAAGCGAATTTTAAAATTCGCCACAACGTGGAGGATGGCAGCGTGCAGCTGGCTGATCACTACCAGCAAAACACTCCAATCGGTGATGGTCCTGTTCTGCTGCCAGACAATCACTATCTGAGCACGCAAAGCGTTCTGTCTAAAGATCCGAACGAGAAACGCGATCATATGGTTCTGCTGGAGTTCGTAACCGCAGCGGGCATCACGCATGGTATGGATGAACTGTACAAAGGCAGCCATCATCATCATCATCACTAA

MSK-ELP(60x amber)-sfGFP-His

ATGAGCAAAGGTGGGCCGGGCGTGGGTGTCCCGGGTGGCGGTGTTCCGGGTGCAGGCGTTCCGGGTTAGGGCGTGCCGGGCGGCGGTGTTCCGGGTGCTGGTGTGCCGGGCTAGGGTGTCCCGGGTGGCGGTGTGCCGGGCGCAGGTGTCCCGGGTTAGGGTGTTCCGGGCGGCGGTGTCCCGGGTGCAGGTGTGCCGGGCTAGGGTGTGCCGGGCGGCGGTGTCCCGGGTGCAGGTGTGCCGGGCTAGGGTGTGCCGGGCGTGGGTGTCCCGGGTGGCGGTGTTCCGGGTGCAGGCGTTCCGGGTTAGGGCGTGCCGGGCGGCGGTGTTCCGGGTGCTGGTGTGCCGGGCTAGGGTGTCCCGGGTGGCGGTGTGCCGGGCGCAGGTGTCCCGGGTTAGGGTGTTCCGGGCGGCGGTGTCCCGGGTGCAGGTGTGCCGGGCTAGGGTGTGCCGGGCGGCGGTGTCCCGGGTGCAGGTGTGCCGGGCTAGGGTGTGCCGGGCGTGGGTGTCCCGGGTGGCGGTGTTCCGGGTGCAGGCGTTCCGGGTTAGGGCGTGCCGGGCGGCGGTGTTCCGGGTGCTGGTGTGCCGGGCTAGGGTGTCCCGGGTGGCGGTGTGCCGGGCGCAGGTGTCCCGGGTTAGGGTGTTCCGGGCGGCGGTGTCCCGGGTGCAGGTGTGCCGGGCTAGGGTGTGCCGGGCGGCGGTGTCCCGGGTGCAGGTGTGCCGGGCTAGGGTGTGCCGGGCGTGGGTGTCCCGGGTGGCGGTGTTCCGGGTGCAGGCGTTCCGGGTTAGGGCGTGCCGGGCGGCGGTGTTCCGGGTGCTGGTGTGCCGGGCTAGGGTGTCCCGGGTGGCGGTGTGCCGGGCGCAGGTGTCCCGGGTTAGGGTGTTCCGGGCGGCGGTGTCCCGGGTGCAGGTGTGCCGGGCTAGGGTGTGCCGGGCGGCGGTGTCCCGGGTGCAGGTGTGCCGGGCTAGGGTGTGCCGGGCGTGGGTGTCCCGGGTGGCGGTGTTCCGGGTGCAGGCGTTCCGGGTTAGGGCGTGCCGGGCGGCGGTGTTCCGGGTGCTGGTGTGCCGGGCTAGGGTGTCCCGGGTGGCGGTGTGCCGGGCGCAGGTGTCCCGGGTTAGGGTGTTCCGGGCGGCGGTGTCCCGGGTGCAGGTGTGCCGGGCTAGGGTGTGCCGGGCGGCGGTGTCCCGGGTGCAGGTGTGCCGGGCTAGGGTGTGCCGGGCGTGGGTGTCCCGGGTGGCGGTGTTCCGGGTGCAGGCGTTCCGGGTTAGGGCGTGCCGGGCGGCGGTGTTCCGGGTGCTGGTGTGCCGGGCTAGGGTGTCCCGGGTGGCGGTGTGCCGGGCGCAGGTGTCCCGGGTTAGGGTGTTCCGGGCGGCGGTGTCCCGGGTGCAGGTGTGCCGGGCTAGGGTGTGCCGGGCGGCGGTGTCCCGGGTGCAGGTGTGCCGGGCTAGGGTGTGCCGGGCGTGGGTGTCCCGGGTGGCGGTGTTCCGGGTGCAGGCGTTCCGGGTTAGGGCGTGCCGGGCGGCGGTGTTCCGGGTGCTGGTGTGCCGGGCTAGGGTGTCCCGGGTGGCGGTGTGCCGGGCGCAGGTGTCCCGGGTTAGGGTGTTCCGGGCGGCGGTGTCCCGGGTGCAGGTGTGCCGGGCTAGGGTGTGCCGGGCGGCGGTGTCCCGGGTGCAGGTGTGCCGGGCTAGGGTGTGCCGGGCGTGGGTGTCCCGGGTGGCGGTGTTCCGGGTGCAGGCGTTCCGGGTTAGGGCGTGCCGGGCGGCGGTGTTCCGGGTGCTGGTGTGCCGGGCTAGGGTGTCCCGGGTGGCGGTGTGCCGGGCGCAGGTGTCCCGGGTTAGGGTGTTCCGGGCGGCGGTGTCCCGGGTGCAGGTGTGCCGGGCTAGGGTGTGCCGGGCGGCGGTGTCCCGGGTGCAGGTGTGCCGGGCTAGGGTGTGCCGGGCGTGGGTGTCCCGGGTGGCGGTGTTCCGGGTGCAGGCGTTCCGGGTTAGGGCGTGCCGGGCGGCGGTGTTCCGGGTGCTGGTGTGCCGGGCTAGGGTGTCCCGGGTGGCGGTGTGCCGGGCGCAGGTGTCCCGGGTTAGGGTGTTCCGGGCGGCGGTGTCCCGGGTGCAGGTGTGCCGGGCTAGGGTGTGCCGGGCGGCGGTGTCCCGGGTGCAGGTGTGCCGGGCTAGGGTGTGCCGGGCGTGGGTGTCCCGGGTGGCGGTGTTCCGGGTGCAGGCGTTCCGGGTTAGGGCGTGCCGGGCGGCGGTGTTCCGGGTGCTGGTGTGCCGGGCTAGGGTGTCCCGGGTGGCGGTGTGCCGGGCGCAGGTGTCCCGGGTTAGGGTGTTCCGGGCGGCGGTGTCCCGGGTGCAGGTGTGCCGGGCTAGGGTGTGCCGGGCGGCGGTGTCCCGGGTGCAGGTGTGCCGGGCTAGGGTGTGCCGGGCGTGGGTGTCCCGGGTGGCGGTGTTCCGGGTGCAGGCGTTCCGGGTTAGGGCGTGCCGGGCGGCGGTGTTCCGGGTGCTGGTGTGCCGGGCTAGGGTGTCCCGGGTGGCGGTGTGCCGGGCGCAGGTGTCCCGGGTTAGGGTGTTCCGGGCGGCGGTGTCCCGGGTGCAGGTGTGCCGGGCTAGGGTGTGCCGGGCGGCGGTGTCCCGGGTGCAGGTGTGCCGGGCTAGGGTGTGCCGGGCGTGGGTGTCCCGGGTGGCGGTGTTCCGGGTGCAGGCGTTCCGGGTTAGGGCGTGCCGGGCGGCGGTGTTCCGGGTGCTGGTGTGCCGGGCTAGGGTGTCCCGGGTGGCGGTGTGCCGGGCGCAGGTGTCCCGGGTTAGGGTGTTCCGGGCGGCGGTGTCCCGGGTGCAGGTGTGCCGGGCTAGGGTGTGCCGGGCGGCGGTGTCCCGGGTGCAGGTGTGCCGGGCTAGGGTGTGCCGGGCTGGCTGGGCCCGGGCGGTGGCGGTCGTAAAGGCGAAGAGCTGTTCACTGGTGTCGTCCCTATTCTGGTGGAACTGGATGGTGATGTCAACGGTCATAAGTTTTCCGTGCGTGGCGAGGGTGAAGGTGACGCAACTAATGGTAAACTGACGCTGAAGTTCATCTGTACTACTGGTAAACTGCCGGTACCTTGGCCGACTCTGGTAACGACGCTGACTTATGGTGTTCAGTGCTTTGCTCGTTATCCGGACCATATGAAGCAGCATGACTTCTTCAAGTCCGCCATGCCGGAAGGCTATGTGCAGGAACGCACGATTTCCTTTAAGGATGACGGCACGTACAAAACGCGTGCGGAAGTGAAATTTGAAGGCGATACCCTGGTAAACCGCATTGAGCTGAAAGGCATTGACTTTAAAGAAGACGGCAATATCCTGGGCCATAAGCTGGAATACAATTTTAACAGCCACAATGTTTACATCACCGCCGATAAACAAAAAAATGGCATTAAAGCGAATTTTAAAATTCGCCACAACGTGGAGGATGGCAGCGTGCAGCTGGCTGATCACTACCAGCAAAACACTCCAATCGGTGATGGTCCTGTTCTGCTGCCAGACAATCACTATCTGAGCACGCAAAGCGTTCTGTCTAAAGATCCGAACGAGAAACGCGATCATATGGTTCTGCTGGAGTTCGTAACCGCAGCGGGCATCACGCATGGTATGGATGAACTGTACAAAGGCAGCCATCATCATCATCATCACTAA

MSK-ELP(8x amber)-sfGFP-His

ATGAGCAAAGGTGGGCCGGGCGTGGGTGTCCCGGGCTAGGGTGTTCCGGGTGCAGGCGTTCCGGGTTAGGGCGTGCCGGGCGGCGGTGTTCCGGGTTAGGGTGTGCCGGGCGCTGGTGTCCCGGGTTAGGGTGTGCCGGGCGCAGGTGTCCCGGGTTAGGGTGTTCCGGGCGGCGGTGTCCCGGGTTAGGGTGTGCCGGGCGCAGGTGTGCCGGGCTAGGGTGTCCCGGGTGGCGGTGTGCCGGGCTAGGGTGTGCCGGGCTGGCTGGGCCCGGGCGGTGGCGGTCGTAAAGGCGAAGAGCTGTTCACTGGTGTCGTCCCTATTCTGGTGGAACTGGATGGTGATGTCAACGGTCATAAGTTTTCCGTGCGTGGCGAGGGTGAAGGTGACGCAACTAATGGTAAACTGACGCTGAAGTTCATCTGTACTACTGGTAAACTGCCGGTACCTTGGCCGACTCTGGTAACGACGCTGACTTATGGTGTTCAGTGCTTTGCTCGTTATCCGGACCATATGAAGCAGCATGACTTCTTCAAGTCCGCCATGCCGGAAGGCTATGTGCAGGAACGCACGATTTCCTTTAAGGATGACGGCACGTACAAAACGCGTGCGGAAGTGAAATTTGAAGGCGATACCCTGGTAAACCGCATTGAGCTGAAAGGCATTGACTTTAAAGAAGACGGCAATATCCTGGGCCATAAGCTGGAATACAATTTTAACAGCCACAATGTTTACATCACCGCCGATAAACAAAAAAATGGCATTAAAGCGAATTTTAAAATTCGCCACAACGTGGAGGATGGCAGCGTGCAGCTGGCTGATCACTACCAGCAAAACACTCCAATCGGTGATGGTCCTGTTCTGCTGCCAGACAATCACTATCTGAGCACGCAAAGCGTTCTGTCTAAAGATCCGAACGAGAAACGCGATCATATGGTTCTGCTGGAGTTCGTAACCGCAGCGGGCATCACGCATGGTATGGATGAACTGTACAAAGGCAGCCATCATCATCATCATCACTAA

MSK-ELP(16x amber)-sfGFP-His

ATGAGCAAAGGTGGGCCGGGCGTGGGTGTCCCGGGCTAGGGTGTTCCGGGTGCAGGCGTTCCGGGTTAGGGCGTGCCGGGCGGCGGTGTTCCGGGTTAGGGTGTGCCGGGCGCTGGTGTCCCGGGTTAGGGTGTGCCGGGCGCAGGTGTCCCGGGTTAGGGTGTTCCGGGCGGCGGTGTCCCGGGTTAGGGTGTGCCGGGCGCAGGTGTGCCGGGCTAGGGTGTCCCGGGTGGCGGTGTGCCGGGCTAGGGTGTGCCGGGCGTGGGTGTCCCGGGCTAGGGTGTTCCGGGTGCAGGCGTTCCGGGTTAGGGCGTGCCGGGCGGCGGTGTTCCGGGTTAGGGTGTGCCGGGCGCTGGTGTCCCGGGTTAGGGTGTGCCGGGCGCAGGTGTCCCGGGTTAGGGTGTTCCGGGCGGCGGTGTCCCGGGTTAGGGTGTGCCGGGCGCAGGTGTGCCGGGCTAGGGTGTCCCGGGTGGCGGTGTGCCGGGCTAGGGTGTGCCGGGCTGGCTGGGCCCGGGCGGTGGCGGTCGTAAAGGCGAAGAGCTGTTCACTGGTGTCGTCCCTATTCTGGTGGAACTGGATGGTGATGTCAACGGTCATAAGTTTTCCGTGCGTGGCGAGGGTGAAGGTGACGCAACTAATGGTAAACTGACGCTGAAGTTCATCTGTACTACTGGTAAACTGCCGGTACCTTGGCCGACTCTGGTAACGACGCTGACTTATGGTGTTCAGTGCTTTGCTCGTTATCCGGACCATATGAAGCAGCATGACTTCTTCAAGTCCGCCATGCCGGAAGGCTATGTGCAGGAACGCACGATTTCCTTTAAGGATGACGGCACGTACAAAACGCGTGCGGAAGTGAAATTTGAAGGCGATACCCTGGTAAACCGCATTGAGCTGAAAGGCATTGACTTTAAAGAAGACGGCAATATCCTGGGCCATAAGCTGGAATACAATTTTAACAGCCACAATGTTTACATCACCGCCGATAAACAAAAAAATGGCATTAAAGCGAATTTTAAAATTCGCCACAACGTGGAGGATGGCAGCGTGCAGCTGGCTGATCACTACCAGCAAAACACTCCAATCGGTGATGGTCCTGTTCTGCTGCCAGACAATCACTATCTGAGCACGCAAAGCGTTCTGTCTAAAGATCCGAACGAGAAACGCGATCATATGGTTCTGCTGGAGTTCGTAACCGCAGCGGGCATCACGCATGGTATGGATGAACTGTACAAAGGCAGCCATCATCATCATCATCACTAA

MSK-ELP(24x amber)-sfGFP-His

ATGAGCAAAGGTGGGCCGGGCGTGGGTGTCCCGGGCTAGGGTGTTCCGGGTGCAGGCGTTCCGGGTTAGGGCGTGCCGGGCGGCGGTGTTCCGGGTTAGGGTGTGCCGGGCGCTGGTGTCCCGGGTTAGGGTGTGCCGGGCGCAGGTGTCCCGGGTTAGGGTGTTCCGGGCGGCGGTGTCCCGGGTTAGGGTGTGCCGGGCGCAGGTGTGCCGGGCTAGGGTGTCCCGGGTGGCGGTGTGCCGGGCTAGGGTGTGCCGGGCGTGGGTGTCCCGGGCTAGGGTGTTCCGGGTGCAGGCGTTCCGGGTTAGGGCGTGCCGGGCGGCGGTGTTCCGGGTTAGGGTGTGCCGGGCGCTGGTGTCCCGGGTTAGGGTGTGCCGGGCGCAGGTGTCCCGGGTTAGGGTGTTCCGGGCGGCGGTGTCCCGGGTTAGGGTGTGCCGGGCGCAGGTGTGCCGGGCTAGGGTGTCCCGGGTGGCGGTGTGCCGGGCTAGGGTGTGCCGGGCGTGGGTGTCCCGGGCTAGGGTGTTCCGGGTGCAGGCGTTCCGGGTTAGGGCGTGCCGGGCGGCGGTGTTCCGGGTTAGGGTGTGCCGGGCGCTGGTGTCCCGGGTTAGGGTGTGCCGGGCGCAGGTGTCCCGGGTTAGGGTGTTCCGGGCGGCGGTGTCCCGGGTTAGGGTGTGCCGGGCGCAGGTGTGCCGGGCTAGGGTGTCCCGGGTGGCGGTGTGCCGGGCTAGGGTGTGCCGGGCTGGCTGGGCCCGGGCGGTGGCGGTCGTAAAGGCGAAGAGCTGTTCACTGGTGTCGTCCCTATTCTGGTGGAACTGGATGGTGATGTCAACGGTCATAAGTTTTCCGTGCGTGGCGAGGGTGAAGGTGACGCAACTAATGGTAAACTGACGCTGAAGTTCATCTGTACTACTGGTAAACTGCCGGTACCTTGGCCGACTCTGGTAACGACGCTGACTTATGGTGTTCAGTGCTTTGCTCGTTATCCGGACCATATGAAGCAGCATGACTTCTTCAAGTCCGCCATGCCGGAAGGCTATGTGCAGGAACGCACGATTTCCTTTAAGGATGACGGCACGTACAAAACGCGTGCGGAAGTGAAATTTGAAGGCGATACCCTGGTAAACCGCATTGAGCTGAAAGGCATTGACTTTAAAGAAGACGGCAATATCCTGGGCCATAAGCTGGAATACAATTTTAACAGCCACAATGTTTACATCACCGCCGATAAACAAAAAAATGGCATTAAAGCGAATTTTAAAATTCGCCACAACGTGGAGGATGGCAGCGTGCAGCTGGCTGATCACTACCAGCAAAACACTCCAATCGGTGATGGTCCTGTTCTGCTGCCAGACAATCACTATCTGAGCACGCAAAGCGTTCTGTCTAAAGATCCGAACGAGAAACGCGATCATATGGTTCTGCTGGAGTTCGTAACCGCAGCGGGCATCACGCATGGTATGGATGAACTGTACAAAGGCAGCCATCATCATCATCATCACTAA

MSK-ELP(32x amber)-sfGFP-His

ATGAGCAAAGGTGGGCCGGGCGTGGGTGTCCCGGGCTAGGGTGTTCCGGGTGCAGGCGTTCCGGGTTAGGGCGTGCCGGGCGGCGGTGTTCCGGGTTAGGGTGTGCCGGGCGCTGGTGTCCCGGGTTAGGGTGTGCCGGGCGCAGGTGTCCCGGGTTAGGGTGTTCCGGGCGGCGGTGTCCCGGGTTAGGGTGTGCCGGGCGCAGGTGTGCCGGGCTAGGGTGTCCCGGGTGGCGGTGTGCCGGGCTAGGGTGTGCCGGGCGTGGGTGTCCCGGGCTAGGGTGTTCCGGGTGCAGGCGTTCCGGGTTAGGGCGTGCCGGGCGGCGGTGTTCCGGGTTAGGGTGTGCCGGGCGCTGGTGTCCCGGGTTAGGGTGTGCCGGGCGCAGGTGTCCCGGGTTAGGGTGTTCCGGGCGGCGGTGTCCCGGGTTAGGGTGTGCCGGGCGCAGGTGTGCCGGGCTAGGGTGTCCCGGGTGGCGGTGTGCCGGGCTAGGGTGTGCCGGGCGTGGGTGTCCCGGGCTAGGGTGTTCCGGGTGCAGGCGTTCCGGGTTAGGGCGTGCCGGGCGGCGGTGTTCCGGGTTAGGGTGTGCCGGGCGCTGGTGTCCCGGGTTAGGGTGTGCCGGGCGCAGGTGTCCCGGGTTAGGGTGTTCCGGGCGGCGGTGTCCCGGGTTAGGGTGTGCCGGGCGCAGGTGTGCCGGGCTAGGGTGTCCCGGGTGGCGGTGTGCCGGGCTAGGGTGTGCCGGGCGTGGGTGTCCCGGGCTAGGGTGTTCCGGGTGCAGGCGTTCCGGGTTAGGGCGTGCCGGGCGGCGGTGTTCCGGGTTAGGGTGTGCCGGGCGCTGGTGTCCCGGGTTAGGGTGTGCCGGGCGCAGGTGTCCCGGGTTAGGGTGTTCCGGGCGGCGGTGTCCCGGGTTAGGGTGTGCCGGGCGCAGGTGTGCCGGGCTAGGGTGTCCCGGGTGGCGGTGTGCCGGGCTAGGGTGTGCCGGGCTGGCTGGGCCCGGGCGGTGGCGGTCGTAAAGGCGAAGAGCTGTTCACTGGTGTCGTCCCTATTCTGGTGGAACTGGATGGTGATGTCAACGGTCATAAGTTTTCCGTGCGTGGCGAGGGTGAAGGTGACGCAACTAATGGTAAACTGACGCTGAAGTTCATCTGTACTACTGGTAAACTGCCGGTACCTTGGCCGACTCTGGTAACGACGCTGACTTATGGTGTTCAGTGCTTTGCTCGTTATCCGGACCATATGAAGCAGCATGACTTCTTCAAGTCCGCCATGCCGGAAGGCTATGTGCAGGAACGCACGATTTCCTTTAAGGATGACGGCACGTACAAAACGCGTGCGGAAGTGAAATTTGAAGGCGATACCCTGGTAAACCGCATTGAGCTGAAAGGCATTGACTTTAAAGAAGACGGCAATATCCTGGGCCATAAGCTGGAATACAATTTTAACAGCCACAATGTTTACATCACCGCCGATAAACAAAAAAATGGCATTAAAGCGAATTTTAAAATTCGCCACAACGTGGAGGATGGCAGCGTGCAGCTGGCTGATCACTACCAGCAAAACACTCCAATCGGTGATGGTCCTGTTCTGCTGCCAGACAATCACTATCTGAGCACGCAAAGCGTTCTGTCTAAAGATCCGAACGAGAAACGCGATCATATGGTTCTGCTGGAGTTCGTAACCGCAGCGGGCATCACGCATGGTATGGATGAACTGTACAAAGGCAGCCATCATCATCATCATCACTAA

MSK-ELP(48x amber)-sfGFP-His

ATGAGCAAAGGTGGGCCGGGCGTGGGTGTCCCGGGCTAGGGTGTTCCGGGTGCAGGCGTTCCGGGTTAGGGCGTGCCGGGCGGCGGTGTTCCGGGTTAGGGTGTGCCGGGCGCTGGTGTCCCGGGTTAGGGTGTGCCGGGCGCAGGTGTCCCGGGTTAGGGTGTTCCGGGCGGCGGTGTCCCGGGTTAGGGTGTGCCGGGCGCAGGTGTGCCGGGCTAGGGTGTCCCGGGTGGCGGTGTGCCGGGCTAGGGTGTGCCGGGCGTGGGTGTCCCGGGCTAGGGTGTTCCGGGTGCAGGCGTTCCGGGTTAGGGCGTGCCGGGCGGCGGTGTTCCGGGTTAGGGTGTGCCGGGCGCTGGTGTCCCGGGTTAGGGTGTGCCGGGCGCAGGTGTCCCGGGTTAGGGTGTTCCGGGCGGCGGTGTCCCGGGTTAGGGTGTGCCGGGCGCAGGTGTGCCGGGCTAGGGTGTCCCGGGTGGCGGTGTGCCGGGCTAGGGTGTGCCGGGCGTGGGTGTCCCGGGCTAGGGTGTTCCGGGTGCAGGCGTTCCGGGTTAGGGCGTGCCGGGCGGCGGTGTTCCGGGTTAGGGTGTGCCGGGCGCTGGTGTCCCGGGTTAGGGTGTGCCGGGCGCAGGTGTCCCGGGTTAGGGTGTTCCGGGCGGCGGTGTCCCGGGTTAGGGTGTGCCGGGCGCAGGTGTGCCGGGCTAGGGTGTCCCGGGTGGCGGTGTGCCGGGCTAGGGTGTGCCGGGCGTGGGTGTCCCGGGCTAGGGTGTTCCGGGTGCAGGCGTTCCGGGTTAGGGCGTGCCGGGCGGCGGTGTTCCGGGTTAGGGTGTGCCGGGCGCTGGTGTCCCGGGTTAGGGTGTGCCGGGCGCAGGTGTCCCGGGTTAGGGTGTTCCGGGCGGCGGTGTCCCGGGTTAGGGTGTGCCGGGCGCAGGTGTGCCGGGCTAGGGTGTCCCGGGTGGCGGTGTGCCGGGCTAGGGTGTGCCGGGCGTGGGTGTCCCGGGCTAGGGTGTTCCGGGTGCAGGCGTTCCGGGTTAGGGCGTGCCGGGCGGCGGTGTTCCGGGTTAGGGTGTGCCGGGCGCTGGTGTCCCGGGTTAGGGTGTGCCGGGCGCAGGTGTCCCGGGTTAGGGTGTTCCGGGCGGCGGTGTCCCGGGTTAGGGTGTGCCGGGCGCAGGTGTGCCGGGCTAGGGTGTCCCGGGTGGCGGTGTGCCGGGCTAGGGTGTGCCGGGCGTGGGTGTCCCGGGCTAGGGTGTTCCGGGTGCAGGCGTTCCGGGTTAGGGCGTGCCGGGCGGCGGTGTTCCGGGTTAGGGTGTGCCGGGCGCTGGTGTCCCGGGTTAGGGTGTGCCGGGCGCAGGTGTCCCGGGTTAGGGTGTTCCGGGCGGCGGTGTCCCGGGTTAGGGTGTGCCGGGCGCAGGTGTGCCGGGCTAGGGTGTCCCGGGTGGCGGTGTGCCGGGCTAGGGTGTGCCGGGCTGGCTGGGCCCGGGCGGTGGCGGTCGTAAAGGCGAAGAGCTGTTCACTGGTGTCGTCCCTATTCTGGTGGAACTGGATGGTGATGTCAACGGTCATAAGTTTTCCGTGCGTGGCGAGGGTGAAGGTGACGCAACTAATGGTAAACTGACGCTGAAGTTCATCTGTACTACTGGTAAACTGCCGGTACCTTGGCCGACTCTGGTAACGACGCTGACTTATGGTGTTCAGTGCTTTGCTCGTTATCCGGACCATATGAAGCAGCATGACTTCTTCAAGTCCGCCATGCCGGAAGGCTATGTGCAGGAACGCACGATTTCCTTTAAGGATGACGGCACGTACAAAACGCGTGCGGAAGTGAAATTTGAAGGCGATACCCTGGTAAACCGCATTGAGCTGAAAGGCATTGACTTTAAAGAAGACGGCAATATCCTGGGCCATAAGCTGGAATACAATTTTAACAGCCACAATGTTTACATCACCGCCGATAAACAAAAAAATGGCATTAAAGCGAATTTTAAAATTCGCCACAACGTGGAGGATGGCAGCGTGCAGCTGGCTGATCACTACCAGCAAAACACTCCAATCGGTGATGGTCCTGTTCTGCTGCCAGACAATCACTATCTGAGCACGCAAAGCGTTCTGTCTAAAGATCCGAACGAGAAACGCGATCATATGGTTCTGCTGGAGTTCGTAACCGCAGCGGGCATCACGCATGGTATGGATGAACTGTACAAAGGCAGCCATCATCATCATCATCACTAA

MSK-ELP(64x amber)-sfGFP-His

ATGAGCAAAGGTGGGCCGGGCGTGGGTGTCCCGGGCTAGGGTGTTCCGGGTGCAGGCGTTCCGGGTTAGGGCGTGCCGGGCGGCGGTGTTCCGGGTTAGGGTGTGCCGGGCGCTGGTGTCCCGGGTTAGGGTGTGCCGGGCGCAGGTGTCCCGGGTTAGGGTGTTCCGGGCGGCGGTGTCCCGGGTTAGGGTGTGCCGGGCGCAGGTGTGCCGGGCTAGGGTGTCCCGGGTGGCGGTGTGCCGGGCTAGGGTGTGCCGGGCGTGGGTGTCCCGGGCTAGGGTGTTCCGGGTGCAGGCGTTCCGGGTTAGGGCGTGCCGGGCGGCGGTGTTCCGGGTTAGGGTGTGCCGGGCGCTGGTGTCCCGGGTTAGGGTGTGCCGGGCGCAGGTGTCCCGGGTTAGGGTGTTCCGGGCGGCGGTGTCCCGGGTTAGGGTGTGCCGGGCGCAGGTGTGCCGGGCTAGGGTGTCCCGGGTGGCGGTGTGCCGGGCTAGGGTGTGCCGGGCGTGGGTGTCCCGGGCTAGGGTGTTCCGGGTGCAGGCGTTCCGGGTTAGGGCGTGCCGGGCGGCGGTGTTCCGGGTTAGGGTGTGCCGGGCGCTGGTGTCCCGGGTTAGGGTGTGCCGGGCGCAGGTGTCCCGGGTTAGGGTGTTCCGGGCGGCGGTGTCCCGGGTTAGGGTGTGCCGGGCGCAGGTGTGCCGGGCTAGGGTGTCCCGGGTGGCGGTGTGCCGGGCTAGGGTGTGCCGGGCGTGGGTGTCCCGGGCTAGGGTGTTCCGGGTGCAGGCGTTCCGGGTTAGGGCGTGCCGGGCGGCGGTGTTCCGGGTTAGGGTGTGCCGGGCGCTGGTGTCCCGGGTTAGGGTGTGCCGGGCGCAGGTGTCCCGGGTTAGGGTGTTCCGGGCGGCGGTGTCCCGGGTTAGGGTGTGCCGGGCGCAGGTGTGCCGGGCTAGGGTGTCCCGGGTGGCGGTGTGCCGGGCTAGGGTGTGCCGGGCGTGGGTGTCCCGGGCTAGGGTGTTCCGGGTGCAGGCGTTCCGGGTTAGGGCGTGCCGGGCGGCGGTGTTCCGGGTTAGGGTGTGCCGGGCGCTGGTGTCCCGGGTTAGGGTGTGCCGGGCGCAGGTGTCCCGGGTTAGGGTGTTCCGGGCGGCGGTGTCCCGGGTTAGGGTGTGCCGGGCGCAGGTGTGCCGGGCTAGGGTGTCCCGGGTGGCGGTGTGCCGGGCTAGGGTGTGCCGGGCGTGGGTGTCCCGGGCTAGGGTGTTCCGGGTGCAGGCGTTCCGGGTTAGGGCGTGCCGGGCGGCGGTGTTCCGGGTTAGGGTGTGCCGGGCGCTGGTGTCCCGGGTTAGGGTGTGCCGGGCGCAGGTGTCCCGGGTTAGGGTGTTCCGGGCGGCGGTGTCCCGGGTTAGGGTGTGCCGGGCGCAGGTGTGCCGGGCTAGGGTGTCCCGGGTGGCGGTGTGCCGGGCTAGGGTGTGCCGGGCGTGGGTGTCCCGGGCTAGGGTGTTCCGGGTGCAGGCGTTCCGGGTTAGGGCGTGCCGGGCGGCGGTGTTCCGGGTTAGGGTGTGCCGGGCGCTGGTGTCCCGGGTTAGGGTGTGCCGGGCGCAGGTGTCCCGGGTTAGGGTGTTCCGGGCGGCGGTGTCCCGGGTTAGGGTGTGCCGGGCGCAGGTGTGCCGGGCTAGGGTGTCCCGGGTGGCGGTGTGCCGGGCTAGGGTGTGCCGGGCGTGGGTGTCCCGGGCTAGGGTGTTCCGGGTGCAGGCGTTCCGGGTTAGGGCGTGCCGGGCGGCGGTGTTCCGGGTTAGGGTGTGCCGGGCGCTGGTGTCCCGGGTTAGGGTGTGCCGGGCGCAGGTGTCCCGGGTTAGGGTGTTCCGGGCGGCGGTGTCCCGGGTTAGGGTGTGCCGGGCGCAGGTGTGCCGGGCTAGGGTGTCCCGGGTGGCGGTGTGCCGGGCTAGGGTGTGCCGGGCTGGCTGGGCCCGGGCGGTGGCGGTCGTAAAGGCGAAGAGCTGTTCACTGGTGTCGTCCCTATTCTGGTGGAACTGGATGGTGATGTCAACGGTCATAAGTTTTCCGTGCGTGGCGAGGGTGAAGGTGACGCAACTAATGGTAAACTGACGCTGAAGTTCATCTGTACTACTGGTAAACTGCCGGTACCTTGGCCGACTCTGGTAACGACGCTGACTTATGGTGTTCAGTGCTTTGCTCGTTATCCGGACCATATGAAGCAGCATGACTTCTTCAAGTCCGCCATGCCGGAAGGCTATGTGCAGGAACGCACGATTTCCTTTAAGGATGACGGCACGTACAAAACGCGTGCGGAAGTGAAATTTGAAGGCGATACCCTGGTAAACCGCATTGAGCTGAAAGGCATTGACTTTAAAGAAGACGGCAATATCCTGGGCCATAAGCTGGAATACAATTTTAACAGCCACAATGTTTACATCACCGCCGATAAACAAAAAAATGGCATTAAAGCGAATTTTAAAATTCGCCACAACGTGGAGGATGGCAGCGTGCAGCTGGCTGATCACTACCAGCAAAACACTCCAATCGGTGATGGTCCTGTTCTGCTGCCAGACAATCACTATCTGAGCACGCAAAGCGTTCTGTCTAAAGATCCGAACGAGAAACGCGATCATATGGTTCTGCTGGAGTTCGTAACCGCAGCGGGCATCACGCATGGTATGGATGAACTGTACAAAGGCAGCCATCATCATCATCATCACTAA

The sequences of the stop codon suppression reporter constructs and the amilCP chromoprotein construct are as follows:

His_6_-SUMO-sfGFP(1x amber)-strep reporter construct

ATGGGCAGCAGCCATCATCATCATCATCACGGTTCTGACTCCGAAGTCAATCAAGAAGCTAAGCCAGAGGTCAAGCCAGAAGTCAAGCCTGAGACTCACATCAATTTAAAGGTGTCCGATGGATCTTCAGAGATCTTCTTCAAGATCAAAAAGACCACTCCTCTGCGTCGTCTGATGGAAGCGTTCGCTAAAAGACAGGGTAAGGAAATGGACTCCTTAAGATTCTTGTACGACGGTATTAGAATCCAAGCTGATCAGACCCCTGAAGATTTGGACATGGAGGATAACGATATTATTGAGGCTCATCGCGAACAGATTGGTGGCATGTAGAAAGGCGAAGAGCTGTTCACTGGTGTCGTCCCTATTCTGGTGGAACTGGATGGTGATGTCAACGGTCATAAGTTTTCCGTGCGTGGCGAGGGTGAAGGTGACGCAACTAATGGTAAACTGACGCTGAAGTTCATCTGTACTACTGGTAAACTGCCGGTACCTTGGCCGACTCTGGTAACGACGCTGACTTATGGTGTTCAGTGCTTTGCTCGTTATCCGGACCATATGAAGCAGCATGACTTCTTCAAGTCCGCCATGCCGGAAGGCTATGTGCAGGAACGCACGATTTCCTTTAAGGATGACGGCACGTACAAAACGCGTGCGGAAGTGAAATTTGAAGGCGATACCCTGGTAAACCGCATTGAGCTGAAAGGCATTGACTTTAAAGAAGACGGCAATATCCTGGGCCATAAGCTGGAATACAATTTTAACAGCCACAATGTTTACATCACCGCCGATAAACAAAAAAATGGCATTAAAGCGAATTTTAAAATTCGCCACAACGTGGAGGATGGCAGCGTGCAGCTGGCTGATCACTACCAGCAAAACACTCCAATCGGTGATGGTCCTGTTCTGCTGCCAGACAATCACTATCTGAGCACGCAAAGCGTTCTGTCTAAAGATCCGAACGAGAAACGCGATCATATGGTTCTGCTGGAGTTCGTAACCGCAGCGGGCATCACGCATGGTATGGATGAACTGTACAAAAGCGCTTGGAGCCACCCGCAGTTCGAAAAATAA

His_6_-SUMO-sfGFP(3x amber)-strep reporter construct

ATGGGCAGCAGCCATCATCATCATCATCACGGTTCTGACTCCGAAGTCAATCAAGAAGCTAAGCCAGAGGTCAAGCCAGAAGTCAAGCCTGAGACTCACATCAATTTAAAGGTGTCCGATGGATCTTCAGAGATCTTCTTCAAGATCAAAAAGACCACTCCTCTGCGTCGTCTGATGGAAGCGTTCGCTAAAAGACAGGGTAAGGAAATGGACTCCTTAAGATTCTTGTACGACGGTATTAGAATCCAAGCTGATCAGACCCCTGAAGATTTGGACATGGAGGATAACGATATTATTGAGGCTCATCGCGAACAGATTGGTGGCATGTAGAAAGGCGAAGAGCTGTTCACTGGTGTCGTCCCTATTCTGGTGGAACTGGATGGTGATGTCAACGGTCATAAGTTTTCCGTGCGTGGCGAGGGTGAAGGTGACGCAACTTAGGGTAAACTGACGCTGAAGTTCATCTGTACTACTGGTAAACTGCCGGTACCTTGGCCGACTCTGGTAACGACGCTGACTTATGGTGTTCAGTGCTTTGCTCGTTATCCGGACCATATGAAGCAGCATGACTTCTTCAAGTCCGCCATGCCGGAAGGCTATGTGCAGGAACGCACGATTTCCTTTTAGGATGACGGCACGTACAAAACGCGTGCGGAAGTGAAATTTGAAGGCGATACCCTGGTAAACCGCATTGAGCTGAAAGGCATTGACTTTAAAGAAGACGGCAATATCCTGGGCCATAAGCTGGAATACAATTTTAACAGCCACAATGTTTACATCACCGCCGATAAACAAAAAAATGGCATTAAAGCGAATTTTAAAATTCGCCACAACGTGGAGGATGGCAGCGTGCAGCTGGCTGATCACTACCAGCAAAACACTCCAATCGGTGATGGTCCTGTTCTGCTGCCAGACAATCACTATCTGAGCACGCAAAGCGTTCTGTCTAAAGATCCGAACGAGAAACGCGATCATATGGTTCTGCTGGAGTTCGTAACCGCAGCGGGCATCACGCATGGTATGGATGAACTGTACAAAAGCGCTTGGAGCCACCCGCAGTTCGAAAAATAA

His_6_-SUMO-sfGFP(5x amber)-strep reporter construct

ATGGGCAGCAGCCATCATCATCATCATCACGGTTCTGACTCCGAAGTCAATCAAGAAGCTAAGCCAGAGGTCAAGCCAGAAGTCAAGCCTGAGACTCACATCAATTTAAAGGTGTCCGATGGATCTTCAGAGATCTTCTTCAAGATCAAAAAGACCACTCCTCTGCGTCGTCTGATGGAAGCGTTCGCTAAAAGACAGGGTAAGGAAATGGACTCCTTAAGATTCTTGTACGACGGTATTAGAATCCAAGCTGATCAGACCCCTGAAGATTTGGACATGGAGGATAACGATATTATTGAGGCTCATCGCGAACAGATTGGTGGCATGTAGAAAGGCGAAGAGCTGTTCACTGGTGTCGTCCCTATTCTGGTGGAACTGGATGGTGATGTCAACGGTCATAAGTTTTCCGTGCGTGGCGAGGGTGAAGGTGACGCAACTTAGGGTAAACTGACGCTGAAGTTCATCTGTACTACTGGTAAACTGCCGGTACCTTGGCCGACTCTGGTAACGACGCTGACTTATGGTGTTCAGTGCTTTGCTCGTTATCCGGACCATATGAAGCAGCATGACTTCTTCAAGTCCGCCATGCCGGAAGGCTATGTGCAGGAACGCACGATTTCCTTTTAGGATGACGGCACGTACAAAACGCGTGCGGAAGTGAAATTTGAAGGCGATACCCTGGTAAACCGCATTGAGCTGAAAGGCATTGACTTTAAATAGGACGGCAATATCCTGGGCCATAAGCTGGAATACAATTTTAACAGCCACAATGTTTACATCACCGCCGATAAACAAAAAAATGGCATTAAAGCGAATTTTAAAATTCGCCACAACGTGGAGGATGGCAGCGTGCAGCTGGCTGATCACTACCAGCAAAACACTCCAATCGGTTAGGGTCCTGTTCTGCTGCCAGACAATCACTATCTGAGCACGCAAAGCGTTCTGTCTAAAGATCCGAACGAGAAACGCGATCATATGGTTCTGCTGGAGTTCGTAACCGCAGCGGGCATCACGCATGGTATGGATGAACTGTACAAAAGCGCTTGGAGCCACCCGCAGTTCGAAAAATAA

amilCP(Y63amber)-his

ATGAGTGTAATCGCTAAACAAATGACCTACAAGGTTTATATGTCAGGCACGGTCAATGGACACTACTTTGAGGTCGAAGGCGATGGAAAAGGTAAGCCCTACGAGGGGGAGCAGACGGTAAAGCTCACTGTCACCAAGGGCGGACCTCTGCCATTTGCTTGGGATATTTTATCACCACAGTGTCAGTAGGGAAGCATACCATTCACCAAGTACCCTGAAGACATCCCTGACTATGTAAAGCAGTCATTCCCGGAGGGCTATACATGGGAGAGGATCATGAACTTTGAAGATGGTGCAGTGTGTACTGTCAGCAATGATTCCAGCATCCAAGGCAACTGTTTCATCTACCATGTCAAGTTCTCTGGTTTGAACTTTCCTCCCAATGGACCTGTCATGCAGAAGAAGACACAGGGCTGGGAACCCAACACTGAGCGTCTCTTTGCACGAGATGGAATGCTGCTAGGAAACAACTTTATGGCTCTGAAGTTAGAAGGAGGCGGTCACTATTTGTGTGAATTTAAAACTACTTACAAGGCAAAGAAGCCTGTGAAGATGCCAGGGTATCACTATGTTGACCGCAAACTGGATGTAACCAATCACAACAAGGATTACACTTCGGTTGAGCAGTGTGAAATTTCCATTGCACGCAAACCTGTGGTCGCCGGCAGCCATCATCATCATCATCACTAA

# Supplementary References

Baumann, T., Hauf, M., Richter, F., Albers, S., Möglich, A., Ignatova, Z., et al. (2019). Computational aminoacyl-tRNA synthetase library design for photocaged tyrosine. *Int. J. Mol. Sci.* 20. doi:10.3390/ijms20092343.

Budisa, N., Steipe, B., Demange, P., Eckerskorn, C., Kellermann, J., and Huber, R. (1995). High-level Biosynthetic Substitution of Methionine in Proteins by its Analogs 2-Aminohexanoic Acid, Selenomethionine, Telluromethionine and Ethionine in Escherichia coli. *Eur. J. Biochem.* 230, 788–796. doi:10.1111/j.1432-1033.1995.0788h.x.

Meyer, D. E., and Chilkoti, A. (2002). Genetically encoded synthesis of protein-based polymers with precisely specified molecular weight and sequence by recursive directional ligation: Examples from the the elastin-like polypeptide system. *Biomacromolecules* 3, 357–367. doi:10.1021/bm015630n.

Schultz, K. C., Supekova, L., Ryu, Y., Xie, J., Perera, R., and Schultz, P. G. (2006). A genetically encoded infrared probe. *J. Am. Chem. Soc.* 128, 13984–13985. doi:10.1021/ja0636690.

Young, D. D., Young, T. S., Jahnz, M., Ahmad, I., Spraggon, G., and Schultz, P. G. (2011). An evolved aminoacyl-tRNA synthetase with atypical polysubstrate specificity. *Biochemistry* 50, 1894–1900. doi:10.1021/bi101929e.
